# Tissue-specific sex differences in pediatric and adult immune cell composition and function

**DOI:** 10.3389/fimmu.2024.1373537

**Published:** 2024-05-15

**Authors:** Mahina Tabassum Mitul, Jenna M. Kastenschmidt, Suhas Sureshchandra, Zachary W. Wagoner, Andrew M. Sorn, David R. Mcllwain, Jenny E. Hernandez-Davies, Aarti Jain, Rafael de Assis, Douglas Trask, D. Huw Davies, Lisa E. Wagar

**Affiliations:** ^1^ Department of Physiology & Biophysics, University of California, Irvine, Irvine, CA, United States; ^2^ Institute for Immunology, University of California, Irvine, Irvine, CA, United States; ^3^ Center for Virus Research, University of California, Irvine, Irvine, CA, United States; ^4^ Vaccine Research and Development Center, University of California, Irvine, Irvine, CA, United States; ^5^ Department of Microbiology and Immunology, Reno School of Medicine, University of Nevada, Reno, NV, United States; ^6^ Department of Otolaryngology-Head and Neck Surgery, University of California, Irvine, CA, United States

**Keywords:** influenza vaccine, sex differences, tonsil organoids, adaptive immunity, pediatric immunity

## Abstract

Sex-based differences in immune cell composition and function can contribute to distinct adaptive immune responses. Prior work has quantified these differences in peripheral blood, but little is known about sex differences within human lymphoid tissues. Here, we characterized the composition and phenotypes of adaptive immune cells from male and female ex vivo tonsils and evaluated their responses to influenza antigens using an immune organoid approach. In a pediatric cohort, female tonsils had more memory B cells compared to male tonsils direct ex vivo and after stimulation with live-attenuated but not inactivated vaccine, produced higher influenza-specific antibody responses. Sex biases were also observed in adult tonsils but were different from those measured in children. Analysis of peripheral blood immune cells from *in vivo* vaccinated adults also showed higher frequencies of tissue homing CD4 T cells in female participants. Together, our data demonstrate that distinct memory B and T cell profiles are present in male vs. female lymphoid tissues and peripheral blood respectively and suggest that these differences may in part explain sex biases in response to vaccines and viruses.

## Introduction

Sex-specific factors have a profound impact on the immune response, yet are understudied in biomedical research (1, 2). The interactions between sex and age-related changes in sex-specific factors also play an important role in the risk for disease development and severity, including autoimmune diseases (3, 4) and viral infections (5, 6). Previous work has comprehensively described fundamental differences in the cell composition and phenotype of immune cells in peripheral blood; females have a greater count of total B cells and T cells, a higher CD4:CD8 ratio, and increased Th1 CD4 T cell responses compared to males (7–11). However, sex differences in the composition, phenotype, and function of these and other immune cells have not been investigated in human lymphoid tissues, which is where adaptive immune responses are organized. Sex is also a major predictor of the humoral response and overall protection after vaccination (12–17). Our current understanding of sex bias in vaccine efficacy is mostly based on animal model data and human clinical and epidemiological studies. Female mice develop larger germinal centers (GCs) and higher antibody titers compared to male mice following influenza virus infection (18). Antibodies from female mice also show enhanced specificity and avidity against antigenically drifted influenza strains (19–21). Most human influenza vaccine studies have focused on serum antibody responses pre- and post-vaccination to evaluate sex differences in the vaccine response. Engler et al. reported higher levels of influenza virus-neutralizing antibodies in female adults after vaccination compared to male adults (15) and this difference persists into older age, as evidenced by higher rates of seroconversion and seroprotection in aged women compared to aged men (22). In contrast, no sex-specific differences in influenza virus neutralizing titers were observed in other influenza vaccination studies (23–25). Thus, the biological effect of sex on the quantity and quality of influenza-specific antibodies is currently ambiguous.

Sex steroid hormones and immunomodulatory genes located on the X chromosome are proposed to be the main mechanisms mediating sex differences in the vaccine response (12, 26). Physiological concentrations of estrogen promote influenza-specific antibody production by B cells after vaccination (19, 27). In contrast, testosterone concentration correlates with reduced neutralizing antibody responses (28). Toll-like receptor 7 (TLR7*)*, which is critical for antigen-specific B cell activation and antibody class switch recombination, can escape X chromosome inactivation in female immune cells (29). Accordingly, this escape is associated with greater antibody magnitude and quality in mice in response to inactivated influenza A virus (18). Live attenuated influenza vaccines (LAIV) and inactivated influenza vaccines (IIV) are approved in the United States for the prevention of seasonal influenza virus infection (30) and their safety and efficacy have been rigorously addressed in children (31–34), however most studies did not report their findings by sex.

To address the impact of sex on lymphoid tissue composition and function, we performed an analysis of the cell composition and phenotypes of immune cells residing in ex vivo tonsil tissues from children and adults based on sex assigned at birth. Using an immune organoid approach, we also evaluated the humoral and cellular response to LAIV in male and female children and adults. Our analysis identifies sex differences in immune cells from both tonsil and blood and reveals a heightened LAIV-specific antibody response in organoids derived from female compared to male children. However, sex differences in the adaptive response varied by donor age and influenza vaccine type. Analysis of peripheral blood data from *in vivo* IIV-vaccinated adults revealed no sex-based difference in vaccine-specific antibodies but significant differences in lymphoid tissue homing potential of circulating cells. Overall, our findings reiterate the importance of investigating lymphoid tissues for understanding factors that contribute to variation in human adaptive immunity.

## Results

### Female-derived pediatric tonsils are enriched in memory B cells

Tonsil samples were analyzed from male and female participants from both pediatric and adult cohorts (**Supplementary Table 1**). Sex assigned at birth was derived from donor medical records. We collected pediatric tonsil tissues for analysis (n=40, 19 males and 21 females) through the Cooperative Human Tissue Network (CHTN). Although indications for surgery were not available at the individual patient level, the vast majority of tonsillectomies at the collection site (Vanderbilt University Medical Center) are performed for tonsillar hypertrophy and pathology analysis indicated that tissues were normal. Male and female donors were age-matched (mean 6.1 years for males, 8.2 years for females; not statistically different) as closely as possible to avoid age-related confounders.

**Figure 1:**
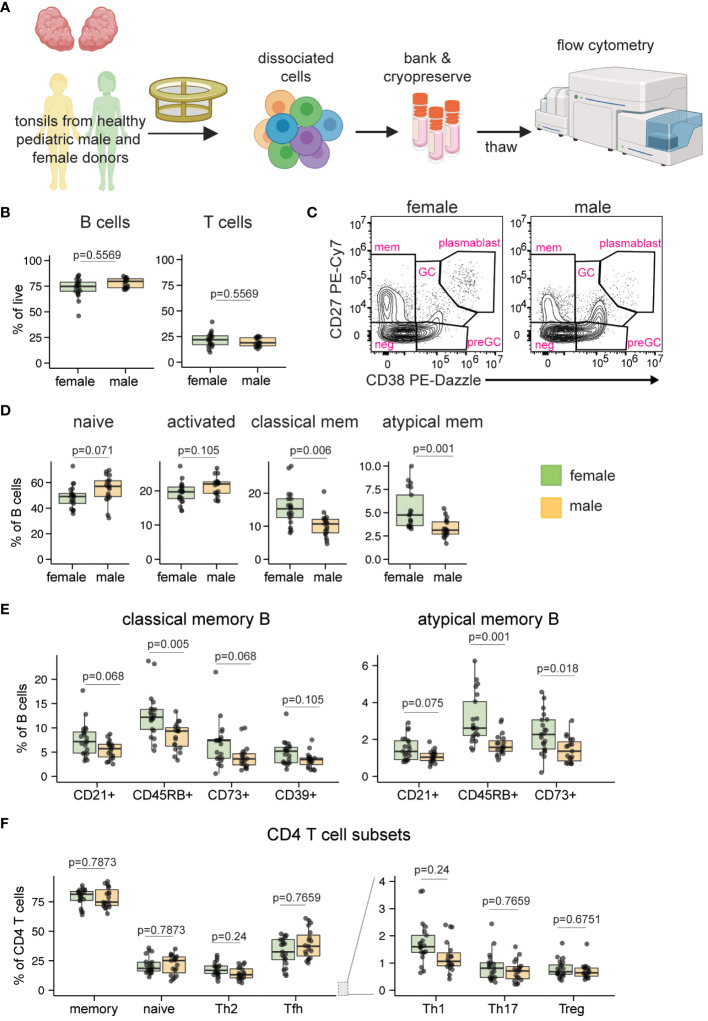
Sex bias in B and T cell phenotypes in ex vivo pediatric tonsils. **(A)** Tonsil samples from pediatric tonsillectomy patients were evaluated for cell composition by flow cytometry. Tonsil tissues from n=40 donors (19 male, 21 female) were assessed. Created with Biorender. **(B)** Frequency of total B and T cells. **(C)** Representative flow cytometry plots showing the distribution of five major B cell subsets. **(D)** Frequencies of naive (IgD+ CD27-), activated (CD83+), classical memory (CD38- CD27+), and atypical memory (IgD- CD27-) B cells. **(E)** Distribution of subset-defining markers in classical and atypical memory B cells. **(F)** Sex-based differences in ex vivo CD4 T cell phenotypes. Each point represents one donor. Mann Whitney U tests followed by multiple hypothesis correction (using the Benjamini & Hochberg method) were used to calculate p values. Boxplots indicate the median value, with hinges denoting the first and third quartiles and whiskers denoting the highest and lowest value within 1.5 times the interquartile range of the hinges.

We first analyzed the cell composition of ex vivo tonsils to identify any sex-based differences within pediatric lymphoid tissues (**Figure 1A**; see **Supplementary Figure 1** for representative gating). The cohort was initially stratified into two age groups (younger children aged 2-9 years and older children aged 10-17 years) to analyze potential effects of puberty on immune cells. However, this analysis indicated that sex differences were similar between younger and older children (data not shown), so all pediatric samples were subsequently analyzed in aggregate. Although the overall proportions of B and T cells were not different based on donor sex (**Figure 1B**), the distribution of phenotypes within these populations differed. Pediatric male donors showed a higher proportion of naïve (p=0.071) and activated (p=0.105) B cells, while pediatric female donors had significantly increased proportions of memory populations, including classical (p=0.006) and atypical (p=0.001) memory B subsets (**Figures 1C, D**). Pre-germinal center, germinal center, and plasmablast B cell phenotypes were not significantly different based on sex (data not shown). Given the female bias in memory B cells and their assumed acquisition as individuals are exposed to antigens over time, we asked whether the difference was consistent across age. A correlation analysis of atypical and classical memory B cells revealed that female-derived tonsils consistently contained more memory B cells, independent of age (**Supplementary Figure 2A**). For both classical and atypical memory B cells, all phenotypes assessed, including CD21+ [associated with high proliferative capacity and antibody production with polyclonal activation (35)], CD45RB+ [inversely correlated with AID expression (36)], CD73+ [suggested to indicate favorable class switch recombination abilities (37)], and CD39+ [which are elevated in tonsils but whose function remains unclear (38)], were elevated in female compared to male tonsils (**Figure 1E**). We also analyzed CD4, CD8, and ɣδ T cell subsets for sex differences. As in tonsil B cells, no differences in the overall frequencies of these cells were noted (**Supplementary Figure 2B**). However, proportionally more Th1 and Th2 CD4 T cells (both p=0.24) were found in pediatric tonsils of females compared to males (**Figure 1F**). An analysis of NK cell frequencies showed no significant difference between male and female tonsils (**Supplementary Figure 2C**). Based on a deep phenotyping analysis of pediatric tonsils, we conclude that female donors possess significantly higher proportions of memory B cells of various phenotypes and slightly elevated Th1 and Th2 T cells compared to male donors of similar age.

### Immune organoids from young male and female tonsils generate distinct B and T cell phenotypes after influenza antigen stimulation

**Figure 2:**
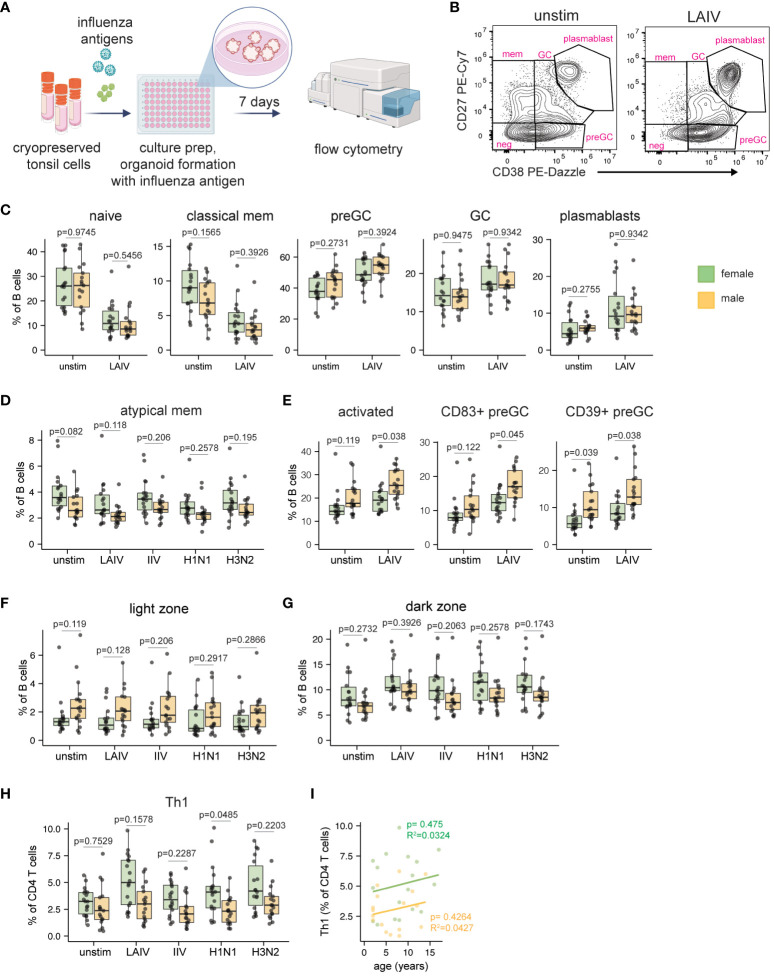
Sex bias in B and T cell responses to influenza antigens in human tonsil organoids. **(A)** Tonsil organoids were prepared from 35 cryopreserved tonsil samples (n=18 female, n=17 male) and stimulated with live-attenuated influenza vaccine (LAIV), inactivated influenza vaccine (IIV), or wild-type H1N1 and H3N2 viruses. Cells were harvested on day 7 for analysis by flow cytometry. Created with Biorender. **(B)** Representative flow cytometry plots showing the distribution of major B cell subsets in unstimulated and LAIV-stimulated tonsil organoids. **(C)** B cell phenotype quantification in male- and female-derived organoids. **(D)** Frequencies of atypical memory B cells (IgD- CD27-) in male and female-derived organoids. **(E)** Frequencies of activated (CD83+) total B cells and activated pre-GC B cells (as measured by CD83 and CD39). Sex differences in the frequency of **(F)** light zone and **(G)** dark zone GC B cells in unstimulated and influenza antigen-stimulated organoids. **(H)** Th1 CD4 T cell frequencies in male and female-derived organoids. **(I)** Linear correlation analysis of Th1 cell frequency and donor age in LAIV-stimulated organoids from male and female participants. Each point represents one donor. Mann Whitney U tests followed by multiple hypothesis correction (using the Benjamini & Hochberg method) were used to calculate p values. Spearman’s rank correlation test and multiple linear regression was performed to calculate the linear regression. Boxplots indicate the median value, with hinges denoting the first and third quartiles and whiskers denoting the highest and lowest value within 1.5 times the interquartile range of the hinges.

The current literature on sex-based differences in influenza vaccine-specific antibody responses in human adults are conflicting (15, 22–25). Although influenza vaccine safety and efficacy are well-established in pediatric populations (31–34), potential sex biases in the magnitude or quality of the vaccine/virus response has not been addressed. Having found sex differences in the immune phenotypes of ex vivo tonsil B cells, we next investigated the impact of sex on the adaptive response to influenza antigens. We used tonsil organoid technology (39, 40) to compare responses to two different influenza vaccines (LAIV and IIV) and two wildtype viruses (H1N1 and H3N2). Dissociated tonsil cells (n=35, 18 female, 17 male) were stimulated with influenza antigens on day 0, cultured into organoids, and B and T cell activation and differentiation were examined on day 7 by flow cytometry (**Figure 2A**). Given that LAIV induced the strongest response (i.e. increase in plasmablast frequency) of the antigens tested (**Supplementary Figure 3A**), we focused the majority of subsequent analyses of sex-based differences on this vaccine format.

Consistent with previous reports (39, 40), LAIV induced strong plasmablast differentiation in most donors’ organoids compared to unstimulated controls (**Supplementary Figure 3B**). Despite differences in starting B cell phenotypes direct ex vivo, organoids from pediatric male and female donors showed similar frequencies of naive, pre-GC, GC, classical, and plasmablast B cells following LAIV stimulation (**Figures 2B, C**). Consistent with our direct ex vivo findings, atypical memory B cells remained elevated (p=0.082 in unstimulated cultures, p=0.118 in LAIV-stimulated cultures) in female- vs. male-derived organoids (**Figure 2D**). We further identified sex influences on B cell activation; male-derived organoids had more B cell activation (CD83+ cells) under both unstimulated (p=0.119) and LAIV-stimulated (p=0.038) conditions (**Figure 2E**). Similarly, preGC B cells were more activated (higher frequency of CD83 and CD39) in male-derived organoids (**Figure 2E**). As previous reports showed that female mice develop larger GCs after influenza virus infection compared to male mice (18), we also examined sex-based differences in GC B cell differentiation and phenotype. Although we did not identify a difference in the total number of GC B cells in male- vs. female-derived organoids, we did observe a clear sex difference in the propensity for light vs. dark zone phenotypes (**Figures 2F, G**). Male-derived organoids had higher proportions of light zone phenotype GC B cells compared to female-derived organoids and this effect was independent of antigen stimulation and format (**Figure 2F**). In contrast, female-derived organoid B cells consistently trended towards a proliferative dark zone B cell phenotype (**Figure 2G**).

T cell proportions, phenotypes, and activation were also quantified from organoids on day 7 post-stimulation. Overall, frequencies of total CD4, CD8 and ɣδ T cells were similar between males and females in response to LAIV (**Supplementary Figure 3C**) and to other influenza antigens (**Supplementary Figure 3D**). Within CD4 T cells, the frequencies of Tfh, Treg, Th2, and Th17 subsets were similar between male- and female-derived organoids (**Supplementary Figure 3E**). However, all influenza antigens (including both vaccines and both viruses) elicited a preferential expansion of Th1 CD4 T cells in female-derived organoids compared to male-derived organoids (**Figure 2H**). The female-biased differentiation of Th1 cells was consistent independent of donor age (**Figure 2I**, **Supplementary Figure 3F**). Taken together, our data establish a sex bias in antigen-independent B cell differentiation and activation as well as an influenza antigen-mediated expansion of Th1 cells in female tissue organoids.

### Sex bias in LAIV-induced influenza-specific antibodies in tonsil organoids

**Figure 3:**
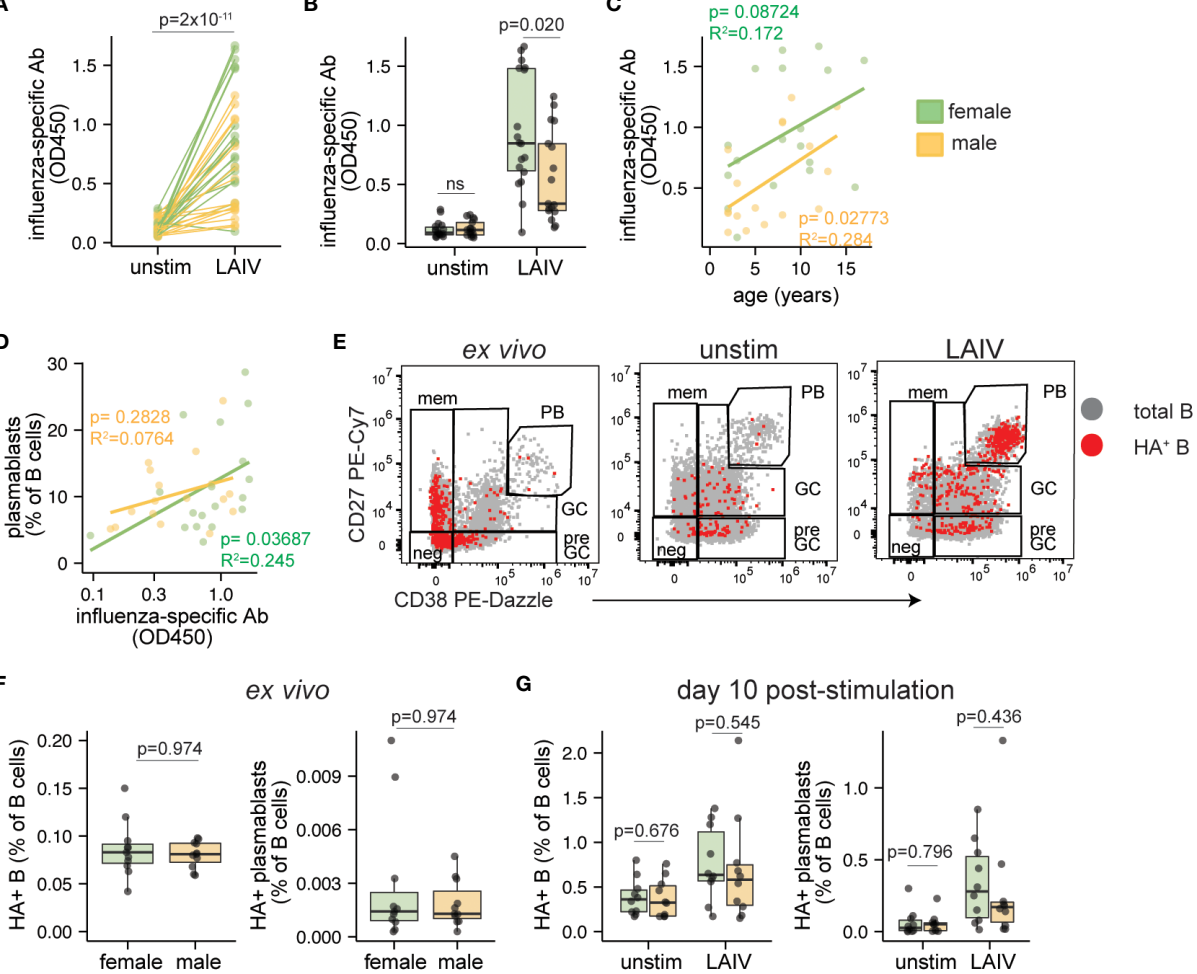
Sex differences in antibody magnitude in tonsil organoids in response to LAIV. Culture supernatants were collected from unstimulated and influenza stimulated tonsil organoids (n=35; 17 male, 18 female) on day 7 to measure influenza-specific antibodies by ELISA. Influenza-specific antibody secretion from unstimulated and LAIV-stimulated organoids on a **(A)** per-donor and **(B)** sex-stratified basis. Correlation between the secreted antibody levels in LAIV-stimulated organoids and **(C)** the age of the tissue donors, **(D)** the frequency of plasmablasts on day 7. **(E)** Representative flow cytometry plots showing A/California 2009 H1N1 HA+ B cells in ex vivo tonsils and day 10 unstimulated or LAIV-stimulated tonsil organoids. **(F)** A/California 2009 H1N1 HA+ B cells and HA+ plasmablasts in ex vivo tonsil tissues (n=20, 10 male, 10 female). **(G)** HA+ B cells and HA+ plasmablasts in unstimulated and LAIV stimulated tonsil organoids on day 10 (n=20; 10 male, 10 female). Mann Whitney U tests were used to calculate p values between groups. Spearman’s rank correlation test and multiple linear regression were performed to calculate linear regression values. Boxplots indicate the median value, with hinges denoting the first and third quartiles and whiskers denoting the highest and lowest value within 1.5 times the interquartile range of the hinges.

We analyzed influenza-specific antibodies and features of influenza-specific B cells in pediatric male- and female-derived tonsil organoids stimulated with influenza vaccines. Organoids stimulated with LAIV effectively elicited an antigen-specific influenza antibody response compared to unstimulated control cultures (**Figure 3A**). In contrast, IIV was poorly immunogenic in most pediatric donor samples (**Supplementary Figure 4A**). Analysis stratified by donor sex showed that influenza-specific antibodies elicited by LAIV were higher (p=0.020) in female- compared to male-derived organoids (**Figure 3B**, **Supplementary Figure 4B**). No sex bias in IIV response was observed (data not shown). The antibody magnitude induced by LAIV was consistent across the entire age range of the pediatric donor cohort (**Figure 3C**). Although influenza-specific antibody secretion weakly correlated with plasmablast frequencies, no sex-based difference in plasmablast differentiation was identified following LAIV stimulation (**Figure 3D**).

Given the significant influence of sex on influenza-specific antibody production, we sought to understand the dynamics of influenza-specific B cells and their phenotypes before and during the response to LAIV. To achieve this, we tracked A/California/2009 H1N1 hemagglutinin (HA)-specific B cells as we and others have done previously (39–41). As expected, most HA+ B cells were of a naive or memory phenotype direct ex vivo; after stimulation, most HA+ B cells acquired pre-GC, GC, and plasmablast phenotypes (**Figure 3E**). The starting proportions of HA+ B cells and HA+ plasmablasts from direct ex vivo tonsils were not different in male and female donors (**Figure 3F**), indicating that the sex difference in organoid antibody responses could not be explained by disparities in the frequency of pre-existing antigen-specific B cells. Although total HA+ B cells and HA+ plasmablasts expanded in organoids after LAIV stimulation (**Supplementary Figures 4C, D**), the extent of their expansion was similar between males and females (**Figure 3G**). Other HA+ B cell phenotypes (such as pre-GC and GC B cells) were also similar between males and females (**Supplementary Figure 4E**). Combined, we showed that LAIV elicited higher levels of influenza-specific antibodies from female organoids compared to male, and this discrepancy is not explained by differences in the starting numbers nor expansion potential of antigen-specific B cells.

### Influenza-specific antibody breadth and neutralization capacity from LAIV-treated organoids are similar in pediatric males and females

**Figure 4:**
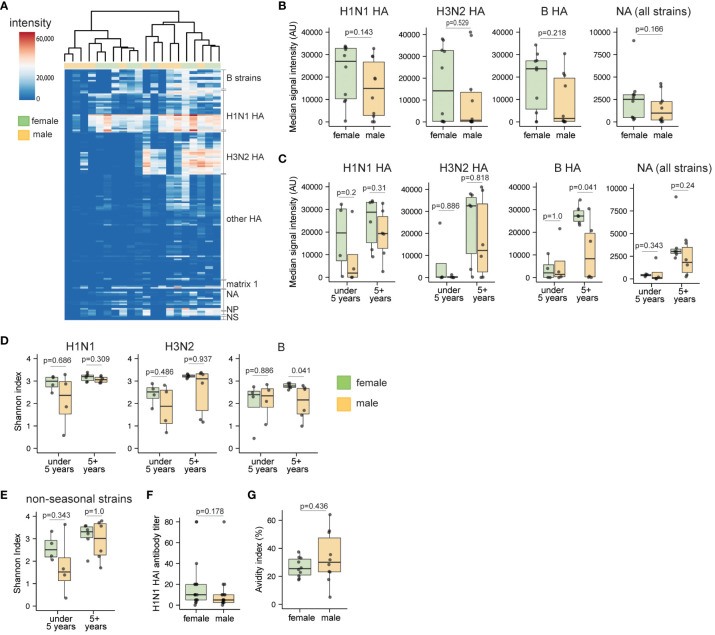
Sex differences in breadth and diversity of influenza-specific antibodies in children in response to LAIV. Culture supernatants from unstimulated and LAIV-stimulated tonsil organoids (n=20, 10 male and 10 female children, age-matched) were collected on day 10 and antibody breadth and diversity were assessed using a high throughput protein microarray. **(A)** Heatmap representing signal intensities for influenza-specific IgG antibodies elicited by LAIV in the tonsil organoids. **(B)** Protein microarray antibody summary data for influenza virus subtypes. Data represent the median signal intensity for antibodies binding all H1N1 HA, H3N2 HA, B HA, and NA proteins from the array. **(C)** Median antibody signal intensities stratified by donor sex and age. Shannon index for influenza-specific IgG antibodies binding **(D)** seasonal and **(E)** non-seasonal virus subtypes. **(F)** A/California/07/2009 H1N1 virus neutralizing antibody titers in LAIV-stimulated tonsil organoids (n=35) from day 7 supernatants. **(G)** Antibody avidity index of LAIV 2019-20 vaccine HA-specific antibodies on day 10 after LAIV stimulation (n=20). Each point is an individual donor. Mann Whitney U tests were performed to determine the statistical significance between groups. Boxplots indicate the median value, with hinges denoting the first and third quartiles and whiskers denoting the highest and lowest value within 1.5 times the interquartile range of the hinges.

Since female-derived organoids demonstrated a capacity to generate more influenza-specific plasmablasts compared to male counterparts, we also investigated whether female-derived organoids also induced broader antibody responses to LAIV. Thus, additional analyses were performed to understand whether sex-based differences in the influenza-specific antibody response were specific to individual virus strains and subtypes, or whether they were broadly elevated in female- compared to male-derived organoids stimulated with LAIV. Using a protein microarray covering 170 influenza proteins derived from human and zoonotic strains over the last century, we compared antibody breadth from organoid culture supernatants from a subset of age-matched male and female samples (**Figure 4A**, **Supplementary Figure 5A**). Consistent with our total influenza-specific antibody analysis of LAIV responses (**Figure 3B**), a trend for higher signal intensity for HA- and neuraminidase (NA)-specific IgG was observed against seasonal viruses in female compared to male-derived organoids (**Supplementary Figure 5B**). To evaluate LAIV-induced antibody breadth, we summarized antibody levels for seasonal HAs and all NAs present on the microarray. Overall, females showed a trend towards higher IgG responses from each of these sub-categories compared to those from males (**Figure 4B**).

Since antibody responses can vary greatly based on age (42) and prior antigen exposure (43), we also assessed the influence of sex on antibody breadth in younger and older children. Influenza-specific antibody responses were generally lower in organoids derived from the under-five group (**Figure 4C**). In the 5+ years group, female-derived organoids generally showed increased influenza HA-specific antibody responses, and this difference was statistically significant for influenza B responses (p=0.041). Further, we calculated the Shannon index as a metric for assessing antibody diversity; overall, the index did not indicate major sex differences in seasonal influenza antibody breadth (**Figure 4D**, **Supplementary Figure 5C**), nor in binding to non-seasonal (e.g., H5 and H7) strains (**Figure 4E**, **Supplementary Figure 5D**). However, in the 5+ years age group, influenza B strain-specific antibody diversity was higher in females compared to males (**Figure 4D**). To investigate potential sex differences in antibody quality, we also measured antibody avidity and virus neutralization from LAIV-stimulated organoids. Hemagglutination inhibition (HAI) titers were similar for culture supernatants from male- and female-derived organoids (**Figure 4F**, **Supplementary Figure 5E**). Although not statistically significant, antibodies from male organoids showed slightly higher avidity (mean 1.3-fold increase) for HA compared to antibodies from female organoids (**Figure 4G**, **Supplementary Figure 5F**). Combined, we conclude that despite higher antibody magnitude in organoids from female donors, influenza-specific antibody breadth and neutralization capabilities are not substantially affected by donor sex.

### Tonsil tissue composition and organoid responses to influenza antigens in adults are also affected by donor sex

**Figure 5:**
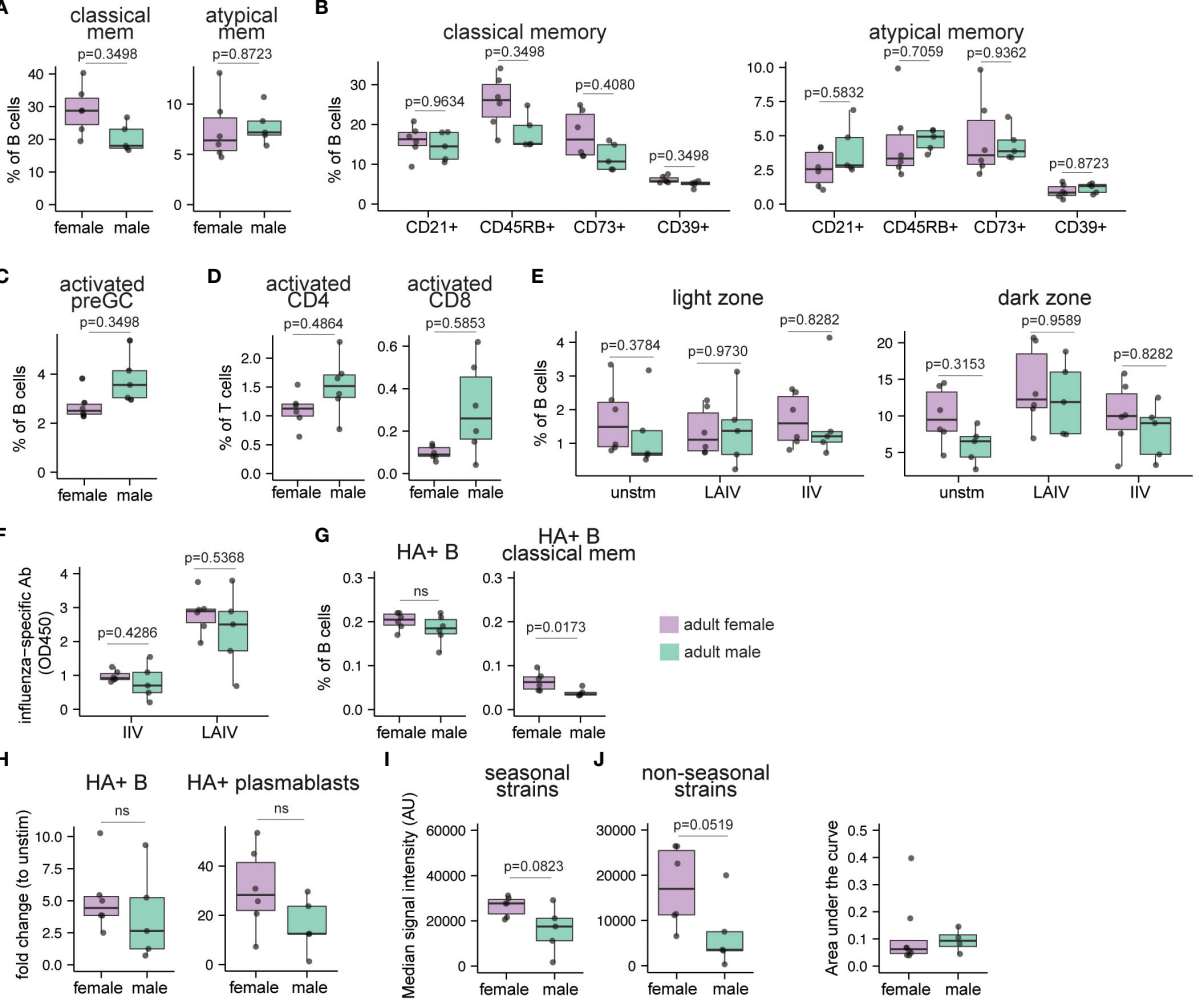
Sex bias in immune cell composition and immune responses to influenza antigens in human tonsil organoids derived from adults. Tonsil samples and influenza antigen stimulated tonsil organoids from age-matched adult males and females (n=11, 5 males, 6 females) were assessed by flow cytometry to evaluate immune cell composition ex vivo and immune response to influenza antigens on day 10. **(A)** cell frequencies of classical memory (CD38- CD27+) and atypical memory (CD27- IgD-) B cells in the ex vivo tonsil tissues. The phenotypes of **(B)** classical memory and atypical memory B cells and **(C)** activated (CD83+) preGC B cells in the ex vivo tonsil tissues. **(D)** Frequencies of activated (HLA-DR+ CD38+) CD4 and CD8 T cells. **(E)** The frequencies of CD83+ light zone and CXCR4+ dark zone GC B cells in tonsil organoids on day 10. **(F)** Influenza-specific antibody secretion from IIV and LAIV-stimulated tonsil organoids. **(G)** Frequencies of A/California/2009 H1N1 HA-specific B cells and classical memory B cells in ex vivo tonsil tissues. **(H)** Fold change in A/California/2009 H1N1 HA-specific B cells and plasmablasts (within total B cells) in LAIV-stimulated tonsil organoids compared to unstimulated organoids. **(I)** Median signal intensities (arbitrary units) for influenza-specific IgG (HA and non-HA) elicited by LAIV in the tonsil organoids on day 10 for all the seasonal and non-seasonal influenza virus subtypes. **(J)** The area under the curve (AUC) for neutralizing antibodies for A/California/07/2009 H1N1 virus present in the culture supernatants in the LAIV-stimulated tonsil organoids derived from adult males and females on day 14 (n=12, 8 females, 4 males). A lower AUC indicates enhanced virus neutralization. Each point represents one donor. Mann Whitney U tests followed by multiple hypothesis correction (using the Benjamini & Hochberg method) were used to calculate p values **(A-E)**. Mann Whitney U tests were used to calculate p values between groups **(F–I)**. Boxplots indicate the median value, with hinges denoting the first and third quartiles and whiskers denoting the highest and lowest value within 1.5 times the interquartile range of the hinges.

To investigate if the sex-based differences we identified in pediatric persons is also present in adults, we next characterized the immune cell composition of ex vivo tonsil tissues in age-matched adults (n=11; 5 males, 6 females; **Supplementary Table 1**). Similar to children, no significant differences in total B and T cell frequencies were observed between adult males and females (**Supplementary Figure 6A**). Further, males and females exhibited comparable frequencies of naive, preGC, GC, and plasmablast B cells (**Supplementary Figure 6B**). Like pediatric females, classical memory B cells were increased (1.43-fold, p=0.35) in female adult compared to male adult tonsils, but there were no significant differences between sexes in atypical memory B cell frequencies (**Figure 5A**; p=0.87). In tonsils from female adults, classical memory B cells with CD45RB (p=0.35) and CD73 (p=0.41) expression were also somewhat higher compared to male donors (**Figure 5B**). In contrast with the pediatric cohort, the proportion of atypical memory B cell subsets were similar in adult males and females (**Figure 5B**). Even though males and females had similar frequencies of total activated B cells (**Supplementary Figure 6C**), the male donors trended towards increased frequencies of activated pre-GC B cells at baseline (**Figure 5C**; p=0.35) as well as increased proportions of activated CD4 (p=0.49) and CD8 (p=0.59) T cells (**Figure 5D**). The total frequencies of CD4 and CD8 T cells (**Supplementary Figure 6D**) and their various subsets (data not shown) were not different based on sex. Based on these data, we conclude that tonsil immune composition is influenced by sex and that distinct sex differences are present in pediatric and adult tissues.

Next, we tested the influence of sex on the adaptive immune response to influenza antigens from adult tonsil tissues using the immune organoid model. We found that overall frequencies of B cell subsets in tonsil organoids were not significantly different between adult males and females in response to LAIV (**Supplementary Figure 6E**). In contrast to pediatric donors, we did not observe a distinct difference in the distribution of light zone and dark zone GC B cells in the unstimulated, LAIV and IIV-stimulated tonsil organoids derived from male and female adults (**Figure 5E**). Assessment of the T cell response from influenza-stimulated organoids did not reveal any significant differences in the total frequencies or phenotypes of CD4 and CD8 T cells between males and females (data ​​not shown).

Several previous studies reported higher *in vivo* humoral immune responses in female compared to male adults after influenza vaccination (15, 22, 28). However, little is known about sex differences in the humoral response to influenza vaccines in children. Therefore, we quantified influenza-specific antibodies in culture supernatants from influenza vaccine-stimulated tonsil organoids by ELISA. As expected, antibody magnitude was higher in adults compared to children (**Figures 3B**, **5F**). However, no significant differences in antibody magnitude were observed between males and females in response to IIV or LAIV (**Figure 5F**). Although starting frequencies of HA-specific total B cells were similar, female tonsils had a higher frequency of HA+ classical memory B cells ex vivo than male tonsils (**Figure 5G**). Moreover, a trend toward increased frequencies of total HA+ B cells and HA+ plasmablasts was observed in the adult female-derived tonsil organoids in response to LAIV compared to males (**Figure 5H**, **Supplementary Figures 6F, G**). We also evaluated potential differences in the breadth and diversity of the influenza-specific antibodies. Compared to males, female organoids made more influenza-specific IgG against seasonal and non-seasonal influenza proteins (**Figure 5I**). Antibody avidity from LAIV-stimulated tonsil organoids was similar between males and females (**Supplementary Figure 6I**). In an independent cohort of adult tonsil organoids, LAIV elicited a stronger neutralizing antibody response in female- compared to male-derived organoids (**Figure 5J**). Collectively, our data indicate an elevated starting pool of HA+ memory B cells in female adult tonsils and greater expansion of these cells likely contributes to increased antibody responses. Female-derived organoids additionally show enhanced antibody breadth and functional neutralization compared to male organoids in response to LAIV.

### The peripheral blood from adult females is enriched in memory and lymphoid-homing T cell subsets

Finally, we sought to understand how sex differences in lymphoid tissues might manifest in peripheral blood after influenza vaccination. We independently analyzed data from a previously published influenza vaccination and virus challenge study to investigate how sex assigned at birth affects immune cell composition in peripheral blood and influenza vaccination outcome *in vivo*. McIlwain et al. performed a mass cytometry analysis of healthy adult volunteers before and after vaccination with IIV (44). Additional measurements of the vaccine response included ELISPOT for antibody-secreting cells, cytokine-secreting T cells and hemagglutination inhibition (HAI) for virus neutralization on day 30 post-vaccination. Sex differences in peripheral blood immune cell composition before and seven days after vaccination were evaluated.

**Figure 6:**
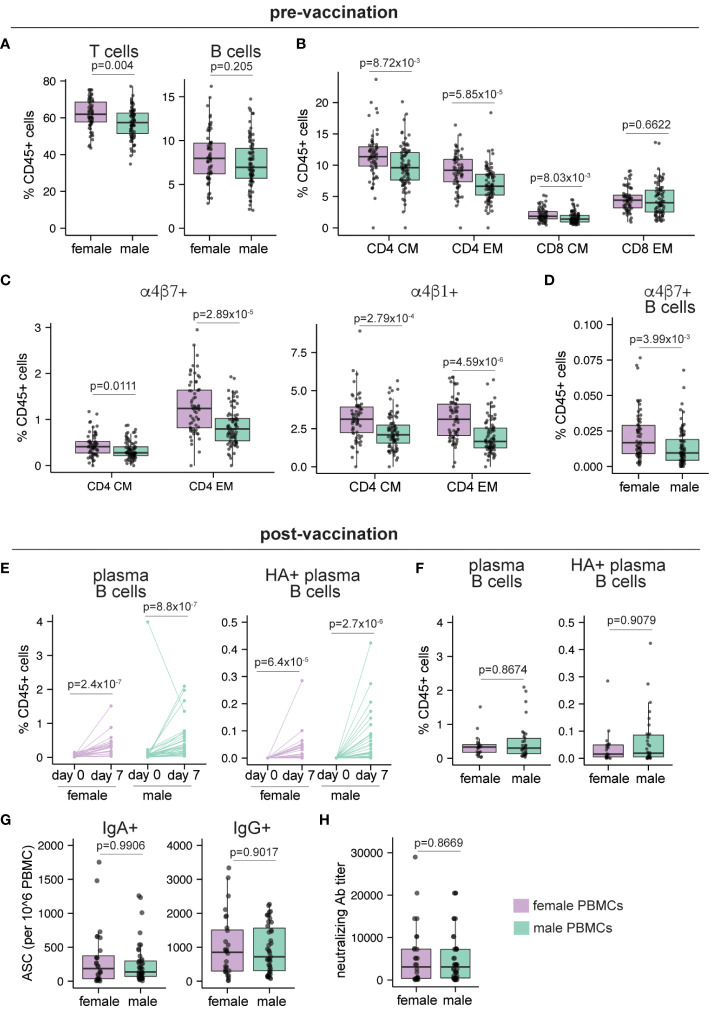
Sex differences in human peripheral blood pre- and post-vaccination. Peripheral blood samples were collected from adult healthy volunteers on day 0 (pre-vaccination, n=149; 65 female, 84 male) and day 7 post IIV vaccination (n=56; 33 male, 23 female). Mass cytometry was used to characterize the immune cells as described in McIlwain et al. Frequencies of **(A)** B and T cells, **(B)** central memory (CM) and effector memory (EM) CD4 and CD8 T cells, **(C)** gut homing (α4β7+) and lymphoid homing (α4β1+) CD4 CM and EM T cells, **(D)** gut homing non-plasma B cells before vaccination. **(E)** Frequencies of plasma B cells and A/California/2009 H1N1 HA-specific plasma B cells before and 7 days after vaccination. **(F)** Frequencies of plasma B cells and A/California/2009 H1N1 HA-specific plasma B cells in female and male donors on day 7 post-vaccination. **(G)** Number of IgA+ and IgG+ antibody secreting cells (ASCs) enumerated by ELISPOT on day 30 post-vaccination (n=71; 28 female, 43 male). **(H)** Neutralizing antibody titers for A/California/2009 H1N1 virus on day 30 post-vaccination (n=71; 28 female, 43 male). Each point represents one donor. Mann Whitney U tests were used to calculate p values. Additionally, multiple hypothesis correction (using the Benjamini & Hochberg method) was performed for data in **(A–D, F)** Significance levels are shown for FDR < 0.1. Boxplots indicate the median value, with hinges denoting the first and third quartiles and whiskers denoting the highest and lowest value within 1.5 times the interquartile range of the hinges.

We first focused on pre-vaccination differences in immune cell composition from peripheral blood. In line with prior literature, the frequency of the T cells (as a proportion of total CD45+ cells) was significantly higher (p=0.004) in females compared to males, while B cell proportions were similar (p=0.205) (**Figure 6A**). Consistent with previous studies (8, 9), females showed higher CD4 T cell proportions (p=6.44x10^-4^) compared to males (**Supplementary Figure 7A**), while higher frequencies of both classical and non-classical monocytes were detected in male PBMCs (**Supplementary Figure 7B**). Within the memory T cell compartment, frequencies of central and effector memory CD4 T cells and central memory CD8 T cells were significantly higher at baseline (p=8.72x10^-3^, 5.85x10^-5^, and 8.03x10^-3^ respectively) in females compared to males (**Figure 6B**). Moreover, compared to males, females showed small but significantly higher frequencies of tissue-homing (α4β7+ and α4β1+) CD4 T cells (**Figure 6C**) and in the frequency of gut-homing (α4β7+) B cells (**Figure 6D**). Overall, our re-analysis of this published data from adult peripheral blood shows that B and T cells from female participants have a higher propensity for migration into tissue sites.

Next, we explored the effect of influenza vaccination on sex differences in peripheral blood immune cells. Although plasma B cells and HA+ plasma B cells significantly increased in both males and females following IIV vaccination (**Figure 6E**), their day 7 post-vaccination frequencies did not differ based on donor sex (**Figure 6F**). Similarly, frequencies of IgA+ and IgG+ antibody-secreting cells (ASCs) on day 30 post vaccination did not differ between males and females (**Figure 6G**). In line with the ASC values and in agreement with prior studies (23–25), neutralizing antibody titers in males and females were not significantly different (**Figure 6H**). Analysis of IFNγ and Granzyme B secreting T cells also showed no difference based on donor sex (**Supplementary Figure 7C**). Similar to baseline values, tissue homing T cell subsets were higher in female donors compared to male donors after vaccination, and their overall frequencies were similar to the pre-vaccination time point (data not shown). Taken together, these data show that female peripheral blood is enriched in memory and tissue homing T and B cells compared to males, but that these differences do not influence the magnitude or quality of the antibody response generated after IIV immunization in adults.

## Discussion

In this study, we evaluated sex differences in the immune cell composition of human tonsils and assessed the humoral and cellular immune responses to LAIV and IIV using a human tonsil organoid platform. Using flow cytometry, we observed that naive and memory B cells were more prevalent in ex vivo tonsil tissue from pediatric males and females, respectively. These observations suggest age-dependent and age-independent sexual dimorphism in lymphoid tissue cell composition. Analysis of the immune response to influenza antigens in tonsil organoids indicated that, independent of the presence or format of influenza antigens, tonsil organoids from pediatric females exhibited a more proliferation-biased GC B cell phenotype compared to those from males in each cohort. Furthermore, introduction of influenza antigens induced more Th1 CD4 T cells in tonsil organoids from pediatric female donors. We demonstrated that female-derived organoids exhibited heightened influenza-specific antibody production to LAIV compared to their male-derived counterparts. Together, this study shows sexual dichotomy in the basal immune cell composition of lymphoid tissues and that female-derived tonsil organoids produce a more robust antibody response to the live attenuated vaccine.

A novel contribution of this study is the revelation that tonsil tissues derived from female children are enriched in both classical and atypical memory B cell subsets compared to similar-aged male donors. A comparably heightened frequency of classical memory B cells was also observed in adult females compared to age-matched adult males, although the number of donors available for study was limited. The increased prevalence of memory B cell subsets among female tonsil tissues may be attributed to preferential differentiation and survival of memory B cells in females compared to males. Although we did not directly measure or modulate estrogen or estrogen receptor expression levels in this study, prior studies have shown that estrogen plays a role in supporting B cell survival, activation and differentiation (45–47). Memory B cells are also known to express BAFF (B cell-activating factor) receptors and rely on BAFF for their survival (48–50) and elevated levels of BAFF have been measured in cord blood from female infants compared to male infants (51). Moreover, BAFF overexpression has been associated with autoimmune diseases (52, 53), which are more prevalent in biological females (4, 54). Indeed, elevated proportions of atypical memory B cells have been documented in chronic infection (55, 56), aged animals (57), and following vaccination (58, 59), and are also detected in patients with systemic lupus erythematosus (60). Consequently, we posit that increased BAFF levels and higher expression of genes facilitating B cell activation, migration, and proliferation may collectively contribute to B cell differentiation toward memory phenotypes in the secondary lymphoid tissues of female donors. We speculate that sex differences in lymphoid tissue composition may affect productive responses to infections. For example, a previous study demonstrated that male children take longer to recover from respiratory infections (61, 62). A greater proportion of memory B cells could confer an advantage in mounting robust humoral immune responses.

Our analysis of whole blood samples from healthy adult volunteers revealed no sex disparity in total B cell frequencies before vaccination. However, higher frequencies of memory CD4 T cells and the greater proportion of these cells with lymphoid and other tissue homing markers in females suggest an enhanced surveillance and migration of T cells in females relative to males. The gene encoding CD27, a canonical marker for memory B and some T cell subsets, although not X-linked, is overexpressed in females (63). Enhancement of CCR1 and CCR5 expression by estrogen (64) further supports our observation and speculation. Consequently, the combined effect of elevated memory B cell subsets in lymphoid tissues and elevated memory CD4 T cell subsets in peripheral blood, as well as the enhanced migration of circulating T cells into lymphoid tissues in females, may collectively contribute to the robustness of humoral responses.

Another major finding from our study is that influenza antigens elicit distinct B and T cell responses in tonsil organoids derived from male and female donors. GC B cells from pediatric females tended to more frequently develop a CXCR4+ dark zone phenotype compared to males. Within the secondary lymphoid tissue, antigen activated B cells undergo significant proliferation during the germinal center response in the dark zone (65). The observed dark zone-biased GC phenotype in pediatric females, irrespective of the presence or the format of influenza antigens in the tonsil organoids, suggests a higher propensity for B cell proliferation, which might culminate in a larger GC. This finding aligns with previous observations of sexual dimorphism in the structure of germinal centers; female mice develop larger germinal centers in response to influenza antigen (18). Furthermore, we observed a generally higher proportion of Th1 CD4 T cells before and after tonsil organoid culture in female compared to male tonsils. CD4 T cells are critical contributors to antiviral responses through the secretion of pro-inflammatory cytokines such as IFNγ and TNFα (66). CD4 T cells also play an important role in the antibody response by supporting GC formation, affinity maturation, and antibody class switching (67, 68). Elevated levels of estrogen and progesterone, as observed during pregnancy, suppress Th1 differentiation in favor of Th2 cells (69, 70) and can lead to vulnerability to respiratory infections. Consequently, influenza antigen-induced Th1 CD4 T cells, along with the augmented surveillance of lymphoid tissues by memory CD4 T cell subsets, can collectively contribute to the heightened adaptive immune response observed in females.

Within each age group examined, tonsil organoids from female donors produced more robust antibody responses after LAIV stimulation compared to male donors. Additionally, adult females exhibited a higher magnitude of influenza-specific antibodies in response to IIV. The relatively low immunogenicity of IIV in children can be explained by a recent report from our group, which established that the humoral immune response to IIV predominantly relies on HA-specific memory B cells in adults (40). In contrast, LAIV is a cold-adapted virus and better mimics a viral infection by providing appropriate innate immune stimuli. TLR-7 plays a critical role in sensing viral pathogens and the gene encoding TLR-7 can escape X chromosome inactivation in immune cells, including in female B cells, monocytes, and plasmacytoid dendritic cells (pDCs) (29). Due to this phenomenon, 50% more TLR7 mRNA transcript has been detected in biallelic B cells (29). Plasma cells have been shown to express more TLR7 than other B cell subsets (71) and thus they may be particularly affected compared to other subsets. Therefore, heightened expression of TLR-7 by immune cells in female lymphoid tissues, coupled with the subsequent stimulation by LAIV, could contribute to the higher influenza-specific antibody magnitude in both pediatric and adult female-derived organoids. In addition, females generate more cross-reactive antibodies and are better protected against distantly related viruses following vaccination (18, 20, 21). Our observations indicate that, in response to LAIV, antibody responses from pediatric females were generally higher across all influenza subtypes measured, including non-seasonal strains. Although total binding antibodies from LAIV stimulation were higher in pediatric female-derived organoids, LAIV only induced more virus neutralizing antibodies in adult females compared to adult males. Enhanced TLR7 signaling leads to enhanced GC B cells, which in turn, enhance SHM and affinity maturation by AID, resulting in a diverse BCR repertoire (72, 73). It is possible that the greater H1N1 virus neutralizing antibodies induced by LAIV in female adults was driven by the engagement of pre-existing somatically hypermutated BCRs from memory B cells, which are limited in pediatric donors.

In conclusion, our analysis revealed sex differences in immune cell composition of tonsil tissues and in the migration potential of immune cells from peripheral blood to lymphoid tissues. We have described a female sex bias in the abundance of memory B cell subsets in secondary lymphoid tissues and this was especially evident in children. Sex-specific differences in the cellular and humoral immune response to influenza antigens were quantified in pediatric and adult tonsils using an organoid approach and showed that organoids from female donors better support a proliferative, dark zone GC B cell phenotype. We also established that female-derived organoids produce an elevated antibody response to LAIV but that this difference was not observed with an inactivated vaccine. Overall, our study provides new data to explain sex differences in the human adaptive immune response from a lymphoid tissue perspective and supports a model for the consideration of age and sex in the future of vaccine design and dosing.

### Limitations of the study

Although tonsil organoids are a useful tool to investigate various aspects of human adaptive immunity, one of their major limitations is the absence of peripheral factors that could influence the adaptive immune response. Sex steroid hormones can positively or negatively correlate with antibody levels after influenza vaccination (19, 27, 28). Although culture media supplements do contain steroid hormones, all organoid cultures were treated with the same hormone levels regardless of donor age and sex. We were also unable to quantify *in vivo* steroid hormone levels from the donors themselves due to sample limitations. Due to technical limitations, we also restricted our antigen-specific B cell analysis to A/California/2009 H1N1 HA-specific B cells. The higher magnitude of influenza-specific antibodies in females could theoretically be driven by a greater pool of pre-existing HA+ B cells from H3N2 and B strains, which were not directly quantified. Another important consideration is that vaccination data was not available from most of the donors used in this study; although we have no reason to believe that a sex bias exists in the cohort’s influenza vaccination or infection history, we cannot exclude differences in pre-existing immunity as a potential factor contributing to differences in the sex biases observed.

## Methods

### Sample collection and preparation of tonsil organoids

Tonsil pairs (n=40) from healthy male (n=19) and female (n=21) children were collected from the Cooperative Human Tissue Network (CHTN) at the Vanderbilt Medical Center (VUMC). Patients underwent tonsillectomy for obstructive sleep apnea or hypertrophy. The age range was from 2-17 years old and the mean ages of the male and female children were not significantly different. The majority of the children (36/40) in this pediatric cohort were prepubertal (below 13 years old) as estimated based on age. After surgery, the tonsil pairs were put in fresh RPMI with antibiotics and, maintaining the cold chain, shipped overnight to the Wagar lab at the University of California Irvine (UCI). After receiving tonsil pairs, they were processed as previously described (39, 40). In brief, the tonsils were mechanically dissociated in Ham’s F12 media with 2% FBS in a metal processing cup. The cell suspensions were then filtered through a 100 μm nylon strainer and subjected to density gradient centrifugation. The purified live tonsillar cells were enumerated and cryopreserved in FBS with 10% DMSO at −140°C until use. The tonsil samples from adult tissue donors (n=11, 6 females, 5 males) were collected from University of California Irvine Medical Center (UCIMC; ethics approved by the UCI IRB #2020-6075) were processed as described above.

To generate the tonsil organoids, cryopreserved aliquots were thawed, enumerated, and plated in ultra-low attachment (ULA) 96 well flat bottom plates followed by influenza antigen (vaccine or virus) addition. The final density of cells was 7.5 x 10^6^ cells/ml in a final volume of 200 μL (1.5 x 10^6^ cells per well). The organoid media was composed of RPMI1640 with glutamax, 10% FBS, 1x non-essential amino acids, 1x sodium pyruvate, 1x penicillin/streptomycin, 1x Normocin, 1x insulin/selenium/transferrin cocktail and 1 μg/mL of human BAFF (produced recombinantly and purified in-house). The concentration of influenza antigens was previously titrated.^2^ A 1/2000 dilution of LAIV 2019-20 (FluMist Quadrivalent, MedImmune) and 1/10000 dilution of IIV 2018-19 (Sanofi Pasteur) was used. For both wildtype A/California/07/2009 H1N1 virus and A/Switzerland/2013 H3N2 virus, 2.5 hemagglutination units (HAU) was added per culture. The cells were incubated at 37°C, 5% CO_2_ and 100μL of fresh organoid media was added every other day by replacing approximately 100μL media per well.

### Flow cytometry

For cell surface characterization of the immune cells in the ex vivo tonsil tissues and organoids, cells were stained with separate B cell (**Supplementary Table 2**) and T cell (**Supplementary Table 3**) panels following established staining protocols. Briefly, thawed cells or harvested organoids (on day 7 or 10) were washed with FACS buffer (PBS + 0.1% BSA, 0.05% sodium azide and 2mM EDTA) and stained with a cocktail of antibodies (Biolegend) in FACS buffer for 30 minutes on ice. The stained cells were washed twice with the FACS buffer and analyzed using a Novocyte quanteon (ACEA) instrument. For assessing the B and T cell response to influenza antigens in organoids, the stained cells were fixed with 2% paraformaldehyde for 15 minutes on ice due to the scale of the experiments. For all other experiments, stained cells were run unfixed.

HA-specific B cells were identified as described previously (40). Briefly, cells were first incubated with biotinylated A/California/07/2009 (H1N1) HA protein (at 2μg/ml) and Fc block (Biolegend) for 30 minutes on ice. Cells were then thoroughly washed with FACS buffer 3-4 times followed by surface staining with antibody cocktails (**Supplementary Table 4**) containing streptavidin-PE and streptavidin-APC for 30 minutes on ice. After staining, the cells were washed as above and analyzed using a Novocyte quanteon (ACEA). HA protein (Immune Technology) was biotinylated according to manufacturer instructions using EZ-link micro NHS-PEG4-biotinylation kit (Thermo Scientific) at 50-fold molar excess. The biotinylated protein was quantified and titrated prior to staining for specific B cells.

### ELISA for influenza-specific antibody

Influenza HA-specific polyclonal antibodies were detected as previously described (39). High protein-binding assay plates were coated with quadrivalent IIV 2018-19 (Sanofi Pasteur) or HA protein matched to the 2019-20 LAIV season (**Supplementary Table 5**) and incubated overnight at 4°C. The final concentration of each of the HA protein in the coating cocktail was 0.5μg/mL. Non-specific binding was blocked by incubating plates in PBS with 1% BSA for 2 hours at room temperature. 100μL of diluted culture supernatants (diluted with PBS) were added to the washed and blocked wells followed by 1 hour incubation at room temperature. Horseradish peroxidase-conjugated anti-human secondary antibodies to IgM/IgG/IgA were used to detect bound antibodies followed by TMB substrate and 1N HCl to stop the reaction. For influenza-specific antibody quantitation, the A450 optical density (OD) was measured using a Cytation5.

### Antibody avidity index

The avidity of the influenza-specific antibodies was detected as previously described (74, 75). ELISA technique was identical to that above with one modification. After the addition sample incubation step, the plate was treated for 5 minutes with PBS or 8M urea (diluted in PBS) followed by extensive washing with PBS/Tween. After this treatment, plates went on to secondary antibody incubation and substrate addition as above. The avidity index was calculated for each sample as the ratio of OD on the urea treated plate divided by the OD of the same sample on the PBS plate.

### Hemagglutination inhibition assay

Hemagglutination inhibition assays (HIA) were performed with small modifications on previously a published protocol (76). Briefly, culture supernatants from tonsil organoids collected on either day 7 or day 10, were diluted (1/2.5) in PBS and diluted with serial 2-fold dilutions. U-bottom and V-bottom 96-well plates were used respectively for H1N1 A/California/2009 and H3N2 A/Switzerland/2013. Wild type viruses were diluted in PBS to a final concentration of 2 HAU per 25uL in each well. The plate was incubated for 30 minutes at room temperature. A 0.75% red blood cell (RBC) solution (turkey RBC for H1N1 and guinea pig RBC for H3N2) was prepared and added to each well followed by incubation at room temperature for an hour. The HIA titer was calculated by identifying the last well in which the RBCs formed a button (for H1N1) or a halo (for H3N2). The reciprocal value of the corresponding dilution represented the HIA titer.

### Microneutralization assay

Microneutralization assays (MN) in this study were performed with modifications from previously described protocols (77). Briefly, Madin-Darby canine kidney (MDCK) cells were maintained in Eagle’s minimum essential medium (EMEM) containing penicillin/streptomycin and 10% heat-inactivated fetal calf serum and cultured at 37°C with 5% CO_2_ in a humid environment. Cells were subcultured when they reached 80–85% confluency and only early passaged cells were used for MN assays. One day prior to assay, MDCK cells were subcultured into flat-bottom 96 well plates at 1.2 × 10^4^ cells/well in 100µL. Organoid culture supernatants were diluted (1/5) in virus growth media (serum-free EMEM containing 0.6% BSA and 1 µg/mL N-p-Tosyl-l-phenylalanine chloromethyl ketone (TPCK)-treated trypsin, then serially diluted (two-fold) in virus growth medium in a separate 96-well plate. A/California/07/09 x A/Puerto Rico/8/1934 reassortant H1N1 virus (BEI NR-44004) was diluted to 50 TCID50 per 50µL in virus growth media and then added to serially diluted supernatants and incubated for 1 h at 37°C, 5% CO_2_. Wells containing only virus and growth media were also prepared to serve as control samples. Following incubation, media from cell monolayers was replaced with the serum-virus mixtures and incubated for an additional 1 h. Serum-virus mixtures were then replaced with 200µL of virus growth media plus 2% FBS and plates were incubated for 48 hours at 37°C. Cells were then fixed in 4% PFA in PBS for 30 min, washed in PBS, and then permeabilized in 0.1% PBS/Triton X-100 at room temperature for 15 min. Cells were washed and blocked in a blocking buffer containing 3% BSA in PBS for 1 hour at room temperature. Influenza virus nucleoprotein (NP) was detected using anti-NP mAbs (Millipore, MAB 8257 and MAB 8258) diluted 1/1000 in blocking buffer, followed by horseradish peroxidase (HRP)-conjugated anti-mouse IgG (KPL, Cat. No. 074-1802) diluted to 1/3000 in blocking buffer. Plates were developed in TMB peroxidase substrate and reactions were quenched using 0.18 M H_2_SO_4_. Assays were quantified in an ELISA plate reader at OD 450 nm using SoftMax Pro 7.1 software.

### Protein microarray

The diversity and cross reactivity of influenza-specific antibodies were calculated using a high throughput protein microarray containing 170 influenza proteins (**Supplementary Table 6**). Day 10 culture supernatants were diluted 1/5 in protein array blocking buffer (GVS) and incubated on arrays overnight at 4°C. Arrays were washed with Tris-buffered saline containing 0.05% Tween 20 (T-TBS) and were incubated for 1.5 h with goat anti-human IgG Qdot 800 (C47091 Invitrogen; 1/400) in blocking buffer. Arrays were washed 3x in T-TBS, 3x in TBS, rinsed in water, and air dried. Images were acquired and intensities were quantified using the ArrayCAM imager and software (Grace Bio-Labs). Data were normalized using a composite of previously described methods (78, 79). Briefly, control spots were normalized using a quantile-based normalization method. Then, the sum of the control spots was calculated. Finally, for each sample, a rescaling factor was calculated by dividing the sum of the normalized control spots by the sum of the control spots. The resulting factor was then multiplied by the reactivity of each spot. The specificities and cross-reactivity of influenza-specific antibodies were analyzed using R. Dendrogram heatmaps were generated using heatmap2. Other visualizations were generated using ggplot2.

### Statistical analysis

All statistical analyses presented in this manuscript were performed in R. For analysis, manually gated cell populations were compared between males and females in both the pediatric and adult cohort. Mann Whitney U tests followed by multiple hypothesis correction (using the Benjamini and Hochberg method) were used to calculate adjusted p values. Where applicable, significance levels of the cell population differences between males and females were indicated by a cutoff of false discovery rate (FDR) < 0.1. In addition, we also performed Mann Whitney U tests without multiple hypothesis correction for cases where multiple variables were not present. Spearman’s rank correlation test and multiple linear regression was performed to calculate the linear regressions shown.

## Data availability statement

The original contributions presented in the study are included in the article/**Supplementary Material**. Further inquiries can be directed to the corresponding author. Data on the *in vivo* influenza vaccination study were previously published (44). Liebowitz *et al*. reports the study design, enrollment criteria and sex, age, and race/ethnicity of the volunteer study cohort for the *in vivo* influenza vaccination study (80).

## Ethics statement

The studies involving locally consented participants were approved by University of California Irvine IRB (protocol #2020-6075) and were conducted in accordance with the local legislation and institutional requirements. Written informed consent for participation in this study was provided by the participants or the participants’ legal guardians/next of kin. Samples provided by the CHTN were collected as surgical discard materials and received not human subjects determination approval.

## Author contributions

MM: Data curation, Formal analysis, Investigation, Methodology, Project administration, Resources, Software, Visualization, Writing – original draft, Writing – review & editing. JK: Data curation, Writing – review & editing. SS: Data curation, Writing – review & editing. ZW: Data curation, Writing – review & editing. AS: Data curation, Resources, Writing – review & editing. DM: Data curation, Writing – review & editing. JH-D: Data curation, Writing – review & editing. AJ: Data curation, Writing – review & editing. RA: Data curation, Writing – review & editing. DT: Resources, Writing – review & editing. DD: Data curation, Writing – review & editing. LW: Conceptualization, Data curation, Funding acquisition, Investigation, Project administration, Supervision, Validation, Visualization, Writing – review & editing, Writing – original draft.
