# Supplementary material for: Tissue-specific sex differences in pediatric and adult immune cell composition and function
Source: Front Immunol. 2024 May 15;15:1373537. doi: 10.3389/fimmu.2024.1373537 (PMC11133680; doi:10.3389/fimmu.2024.1373537)
Supplement: Supplementary file 1 [file DataSheet_1.pdf]

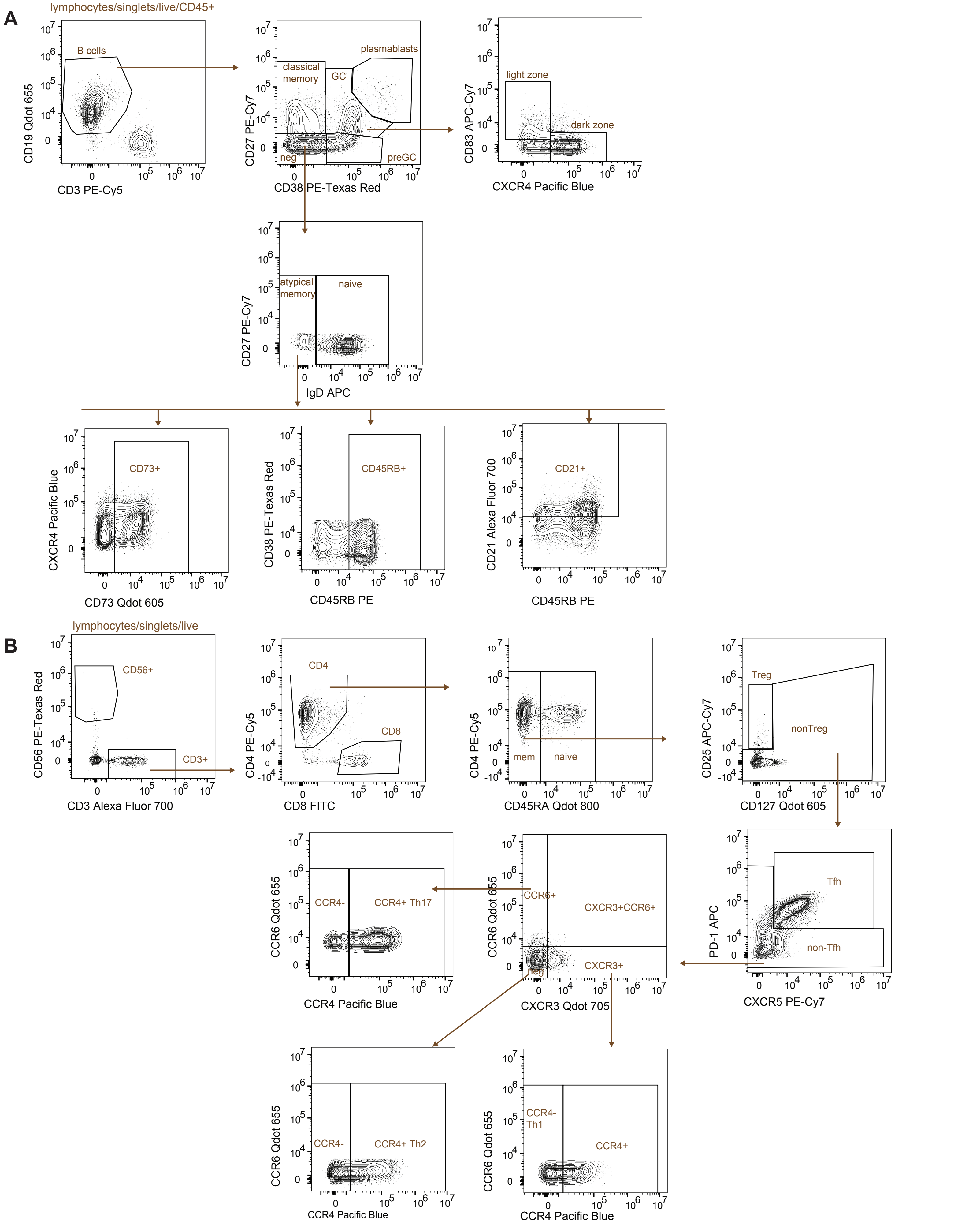

SFig 1: Flow cytometry gating strategy on representative ex vivo tonsil cells. Phenotypes of (**A**) B and (**B**) T cells in tonsil.

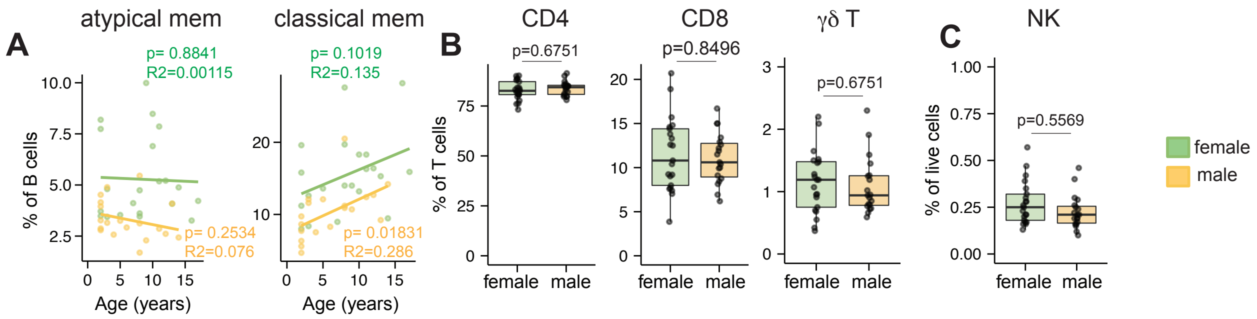

SFig 2: Additional analyses of sex-based differences in *ex vivo* pediatric tonsils. (**A**) Correlation analysis of memory B cell subsets in male and female tissues and donor age. (**B**) Frequencies of CD4, CD8 and γδ T cells. (**C**) Frequency of NK cells among total live cells. Each point represents one donor. Spearman's rank correlation test and multiple linear regression was performed to calculate the linear regression. Mann Whitney U tests followed by multiple hypothesis correction (using the Benjamini & Hochberg method) were used to calculate p values. Boxplots indicate the median value, with hinges denoting the first and third quartiles and whiskers denoting the highest and lowest value within 1.5 times the interquartile range of the hinges.

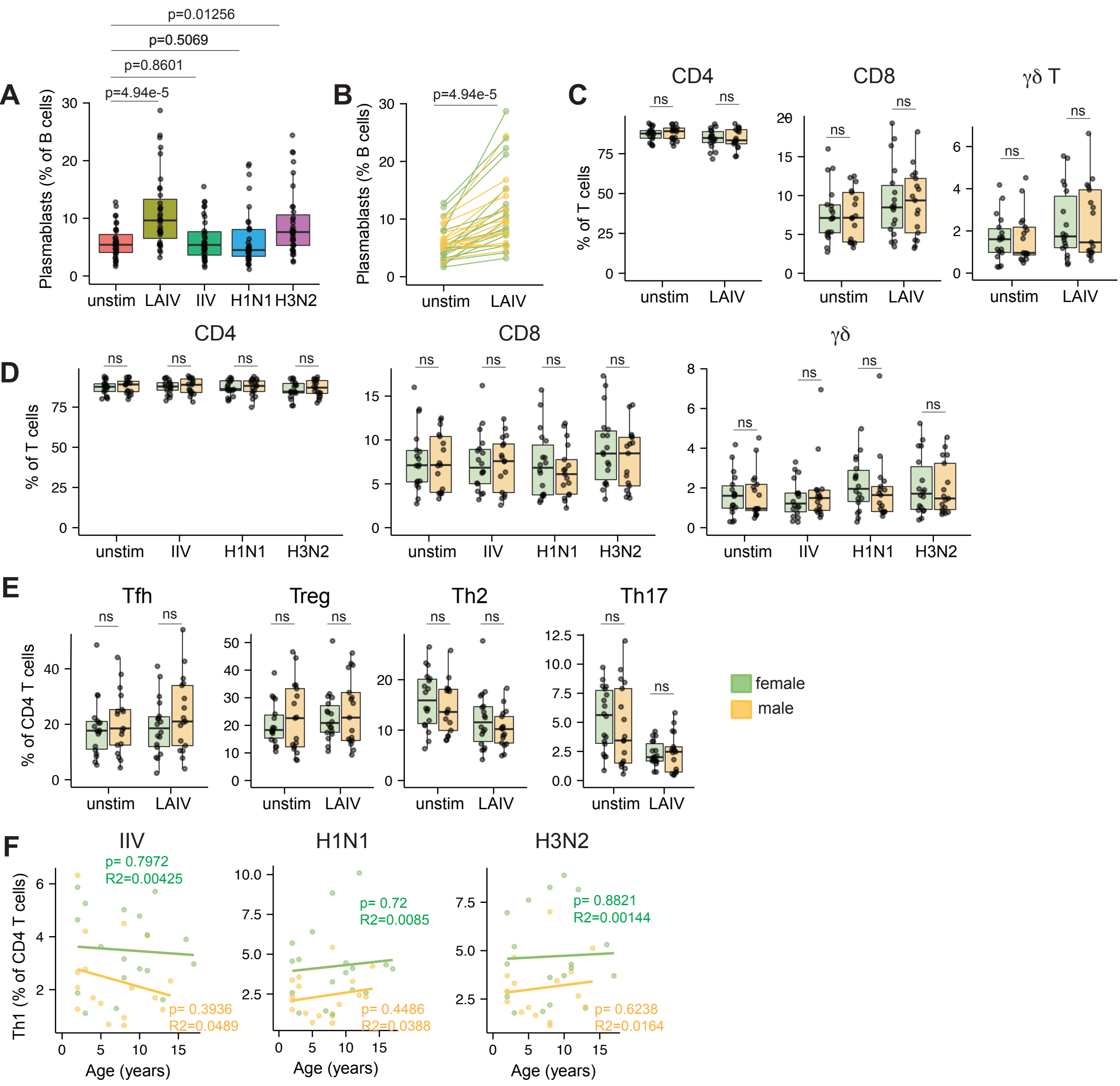

SFig 3: Additional analyses of immune response to influenza antigens in the tonsil organoids. (**A**) The frequencies of plasmablasts in unstimulated, LAIV, IIV vaccine, H1N1 and H3N2 wild type virus-stimulated organoids (n=35, 17 males, 18 females) on day 7. (**B**) Frequencies of plasmablasts in unstimulated and LAIV-stimulated tonsil organoids on per donor basis. The frequencies of CD4, CD8 T and γδ T cells in (**C**) unstimulated and LAIV-stimulated, (D) unstimulated and IIV, H1N1, H3N2 virus-stimulated tonsil organoids on day 7. (**E**) The frequencies of CD4 T cell subsets in the unstimulated and LAIV-stimulated tonsil organoids on day 7. (**F**) Correlation between frequency of Th1 CD4 T cells in IIV, H1N1 and H3N2 virus-stimulated tonsil organoids on day 7 and donor age. Each point represents one donor. Mann Whitney U tests were used to calculate p values between groups (**A-B**). Mann Whitney U tests followed by multiple hypothesis correction (using the Benjamini & Hochberg method) were used to calculate p values (**C-E**). Spearman's rank correlation test and multiple linear regression was performed to calculate the linear regression. Boxplots indicate the median value, with hinges denoting the first and third quartiles and whiskers denoting the highest and lowest value within 1.5 times the interquartile range of the hinges.

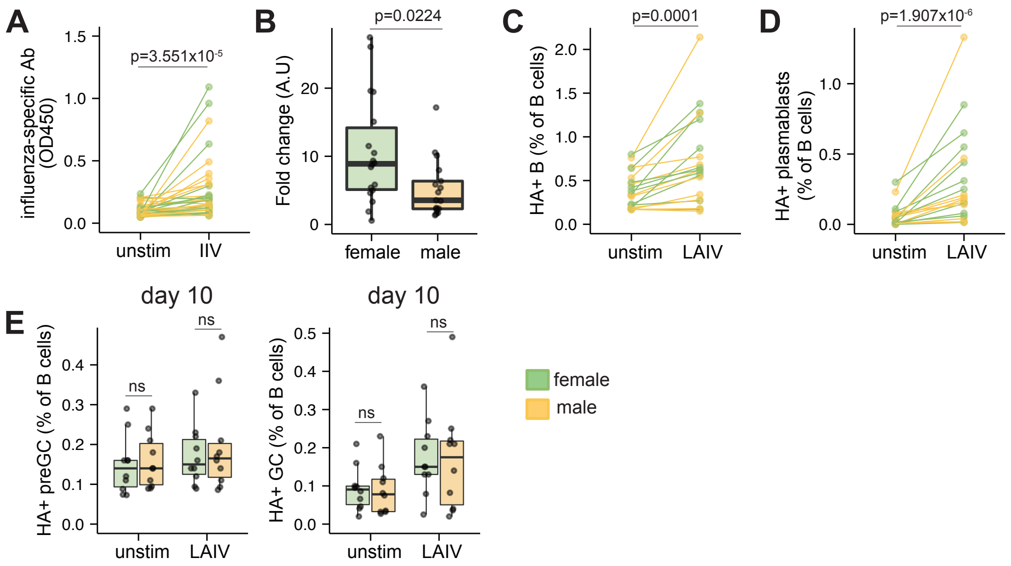

SFig 4: Analysis of antibody magnitude and HA+ B cell phenotype in the tonsil organoids in response to influenza vaccines. (**A**) Influenza-specific antibody secretion from unstimulated and IIV-stimulated organoids (n=35, 17 males, 18 females) on day 7. (**B**) Fold change in influenza-specific antibodies in LAIV-stimulated organoids compared to the unstimulated organoids (n=35, 17 males, 18 females) on day 7. Expansion of A/California/07/2009 H1N1 (**C**) HA+ B cells, (**D**) HA+ plasmablasts, (**E**) HA+ preGC and HA+ GC in the organoids (n=20, 10 males, 10 females) on day 10. Mann Whitney U tests were used to calculate p values between groups. Boxplots indicate the median value, with hinges denoting the first and third quartiles and whiskers denoting the highest and lowest value within 1.5 times the interquartile range of the hinges.

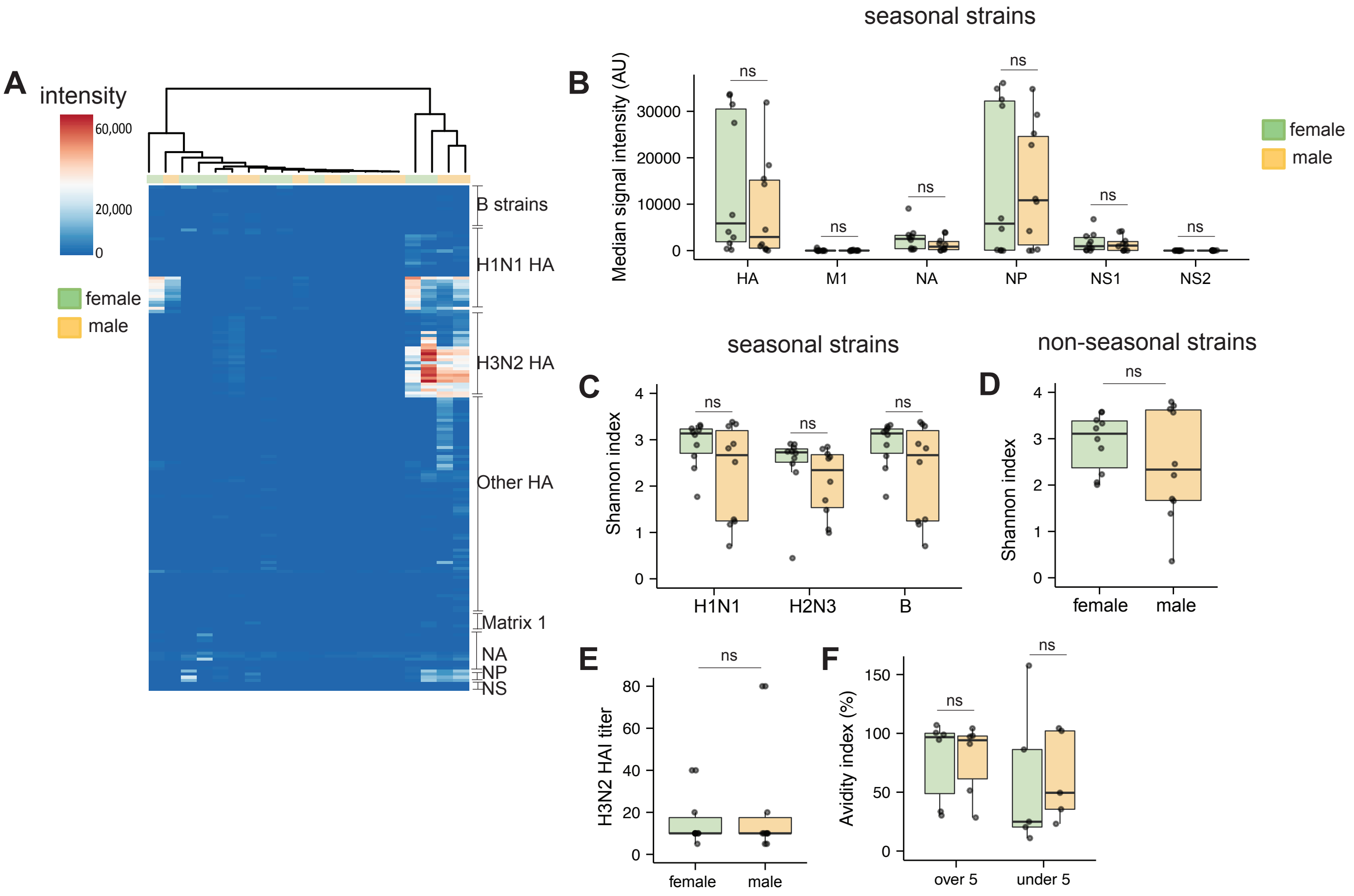

SFig 5: Additional assessment of antibody quantity and quality elicited by LAIV in the tonsil organoids. (**A**) Heatmap representing signal intensities for influenza-specific IgG antibodies in the unstimulated tonsil organoid supernatants on day 10. (**B**) Median signal intensity in males and females in response to LAIV for the influenza proteins from the seasonal virus types. Shannon index for the influenza-specific IgG (HA and non-HA) from the (**C**) seasonal, (**D**) non-seasonal influenza strains. (**E**) A/Switzerland/2013 H3N2 virus neutralizing antibody titers elicited in LAIV-stimulated tonsil organoids on day 10. (**F**) Avidity index for LAIV 2019-20 vaccine HA-specific antibodies in children over and under 5 years old. Each point is an individual donor (n=20). Mann Whitney U tests were performed to determine the statistical significance between groups. Boxplots indicate the median value, with hinges denoting the first and third quartiles and whiskers denoting the highest and lowest value within 1.5 times the interquartile range of the hinges.

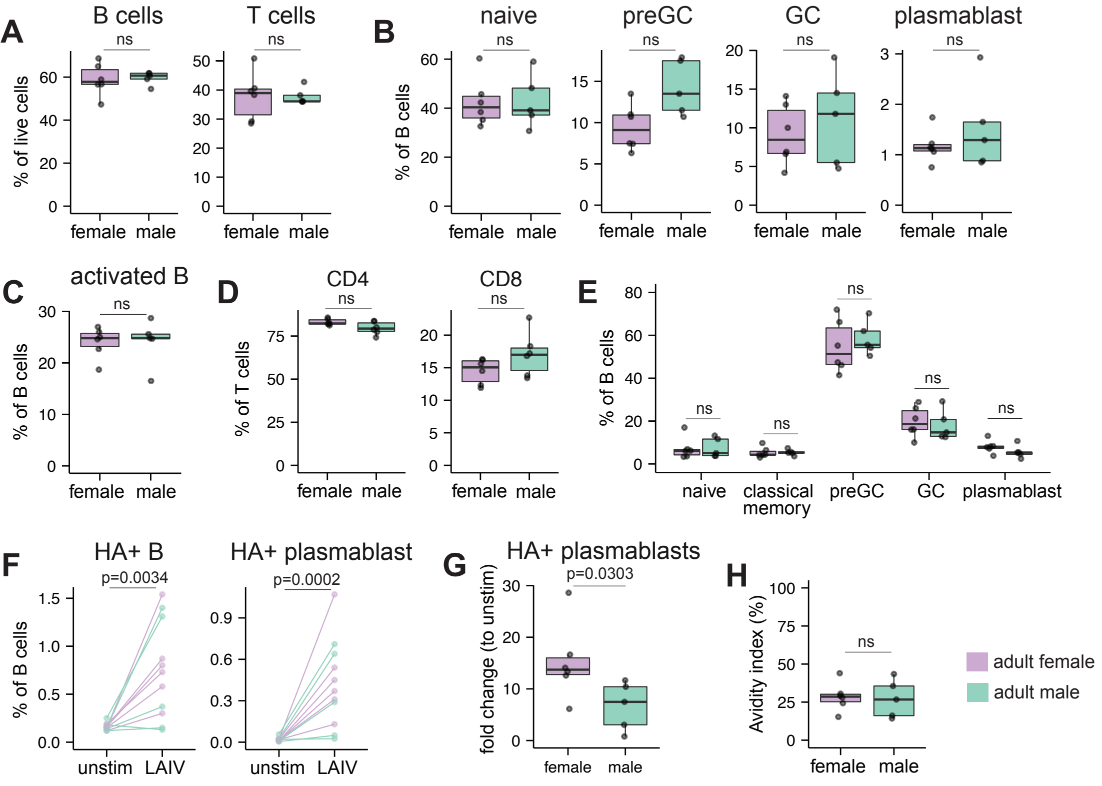

SFig 6: Extended analysis of immune cell composition and immune response to influenza antigens in the tonsil organoids from adult tissue donors. Cell frequencies of (**A**) total B and T cells; (**B**) naive, preGC, GC and plasmablast in the *ex vivo* tonsil tissues (n=11, 6 females, 5 males). Frequencies of (**C**) activated (CD83+) B cells, (**D**) CD4 and CD8 T cells in the *ex vivo* tonsil tissues. (**E**) The frequencies of naive, classical memory, preGC, GC B cells and plasmablasts in LAIV-stimulated tonsil organoids on day 10 (n=11, 6 females, 5 males). Frequencies of A/California/07/2009 H1N1 (**F**) HA+ B cells and HA+ plasmablasts in unstimulated and LAIV-stimulated tonsil organoids on day 10 on per donor basis. (**G**) Fold change in A/California/2009 H1N1 HA-specific plasmablasts (within the plasmablast pool) in LAIV-stimulated tonsil organoids compared to unstimulated organoids on day 10. (**H**) Avidity index of LAIV 2019-20 vaccine HA-specific antibodies in the day 10 culture supernatants from LAIV-stimulated tonsil organoids. Each point represents one donor. Mann Whitney U tests followed by multiple hypothesis corrections were used to calculate p values between groups (**A-E**). Mann Whitney U tests were used to calculate p values between groups (**F-H**). Boxplots indicate the median value, with hinges denoting the first and third quartiles and whiskers denoting the highest and lowest value within 1.5 times the interquartile range of the hinges.

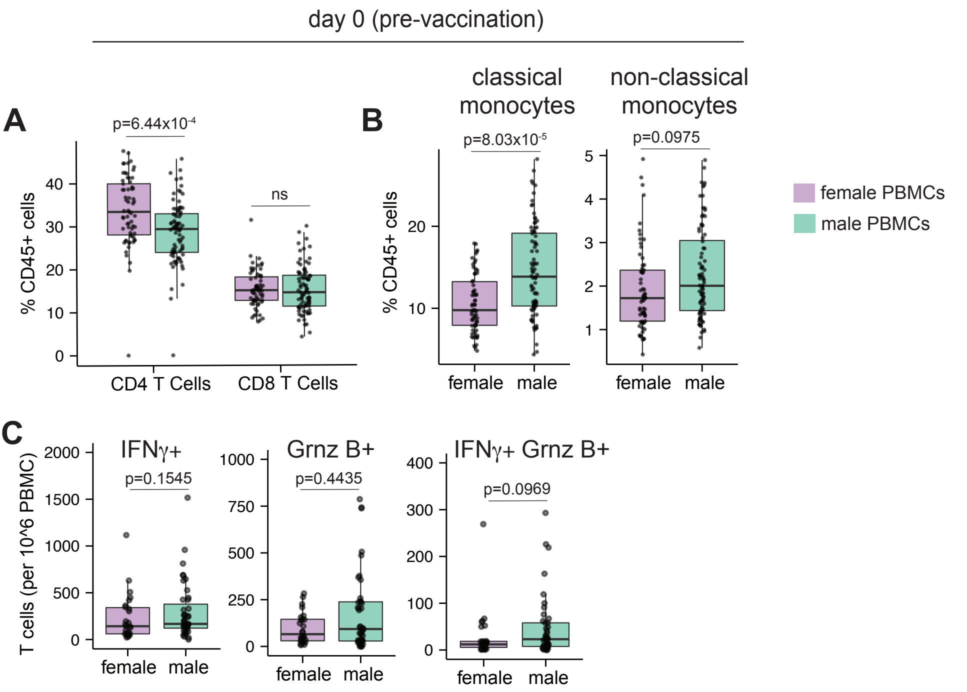

SFig 7: Additional analysis of Sex differences in human peripheral blood pre and post influenza vaccination. The frequencies of (**A**) CD4 and CD8 T cells, (**B**) classical and non-classical monocytes in peripheral blood from healthy adult males and females on day 0. (**C**) Number of IFNγ, granzyme B (Grnz B), and both IFNγ and Grnz B-secreting T cells enumerated by ELISPOT on day 30 post IIV vaccination. Each point represents one donor. Mann Whitney U tests followed by multiple hypothesis correction were used to calculate p values (**A-B**). Significance levels of the cell population differences between males and females are indicated by FDR < 0.1. Mann Whitney U tests were used to calculate p values between groups (**C**). Boxplots indicate the median value, with hinges denoting the first and third quartiles and whiskers denoting the highest and lowest value within 1.5 times the interquartile range of the hinges.
