# Supplementary material for: Tissue-specific sex differences in pediatric and adult immune cell composition and function
Source: Front Immunol. 2024 May 15;15:1373537. doi: 10.3389/fimmu.2024.1373537 (PMC11133680; doi:10.3389/fimmu.2024.1373537)
Supplement: Supplementary file 2 [file DataSheet_2.pdf]

Supplementary table 1. Tonsillectomy donor demographic information. Samples were collected through the Cooperative Human Tissue Network (CHTN) or the UCI Medical Center (UCI). Although donor-specific diagnoses are not available for CHTN samples, the most common indication for surgery at the collection site is tonsillar hypertrophy. Donor age, sex, race, and indication for surgery come from patient medical records.

donor	age (years)	sex	race and/or ethnicity	source	surgical indication
1	8	male	black	CHTN	no data (likely hypertrophy)
2	16	female	white	CHTN	no data (likely hypertrophy)
3	5	female	white	CHTN	no data (likely hypertrophy)
4	2	male	white	CHTN	no data (likely hypertrophy)
5	11	male	white	CHTN	no data (likely hypertrophy)
6	9	male	black	CHTN	no data (likely hypertrophy)
7	2	female	white	CHTN	no data (likely hypertrophy)
8	14	male	white	CHTN	no data (likely hypertrophy)
9	11	female	white	CHTN	no data (likely hypertrophy)
10	12	female	white	CHTN	no data (likely hypertrophy)
11	3	male	black	CHTN	no data (likely hypertrophy)
12	2	female	black	CHTN	no data (likely hypertrophy)
13	11	female	white	CHTN	no data (likely hypertrophy)
14	10	female	black	CHTN	no data (likely hypertrophy)
15	8	female	black	CHTN	no data (likely hypertrophy)
16	2	female	white	CHTN	no data (likely hypertrophy)
17	10	female	white	CHTN	no data (likely hypertrophy)
18	2	male	white	CHTN	no data (likely hypertrophy)
19	9	female	black	CHTN	no data (likely hypertrophy)
20	12	male	white	CHTN	no data (likely hypertrophy)
21	14	female	white	CHTN	no data (likely hypertrophy)
22	2	male	white	CHTN	no data (likely hypertrophy)
23	3	female	white	CHTN	no data (likely hypertrophy)
24	8	male	white	CHTN	no data (likely hypertrophy)
25	5	male	white	CHTN	no data (likely hypertrophy)
26	8	female	white	CHTN	no data (likely hypertrophy)
27	13	female	white	CHTN	no data (likely hypertrophy)
28	13	male	white	CHTN	no data (likely hypertrophy)
29	17	female	black	CHTN	no data (likely hypertrophy)
30	8	male	white	CHTN	no data (likely hypertrophy)
31	3	female	white	CHTN	no data (likely hypertrophy)
32	2	male	white	CHTN	no data (likely hypertrophy)
33	8	female	white	CHTN	no data (likely hypertrophy)
34	6	male	white	CHTN	no data (likely hypertrophy)
35	2	female	white	CHTN	no data (likely hypertrophy)
36	3	male	white	CHTN	no data (likely hypertrophy)
37	4	male	white	CHTN	no data (likely hypertrophy)
38	2	male	white	CHTN	no data (likely hypertrophy)
39	7	female	white	CHTN	no data (likely hypertrophy)
40	2	male	white	CHTN	no data (likely hypertrophy)
UCIMC002	20	female	white, not hispanic/latino	UCI	recurrent tonsillitis
UCIMC012	21	male	white, not hispanic/latino	UCI	recurrent tonsillitis/hypertrophy
UCIMC014	28	female	white, hispanic/latino	UCI	recurrent tonsillitis/hypertrophy
UCIMC029	26	male	white, hispanic/latino	UCI	recurrent tonsillitis/hypertrophy
UCIMC015	20	female	white, not hispanic/latino	UCI	recurrent tonsillitis
UCIMC026	19	male	white, hispanic/latino	UCI	recurrent tonsillitis
UCIMC013	23	female	white, hispanic/latino	UCI	recurrent tonsillitis/hypertrophy
UCIMC023	22	male	asian, hispanic/latino	UCI	recurrent tonsillitis
UCIMC028	34	female	asian, not hispanic/latino	UCI	recurrent tonsillitis/hypertrophy
UCIMC011	37	male	white, hispanic/latino	UCI	hypertrophy
UCIMC006	25	female	white, hispanic/latino	UCI	recurrent tonsillitis

Supplementary table 2: Flow cytometry panel for characterizing the B cells			
**Cell surface Marker**	**Fluorochrome**	**Catalog no.**	**Vendor**
CD138	FITC	356508	Biolegend
CD45	PerCP-Cy5.5	368504	Biolegend
IgD	APC	348222	Biolegend
CD21	AF700	354918	Biolegend
CD83	APC-Cy7	305330	Biolegend
CXCR4	BV421	306518	Biolegend
Zombie	Amcyan	423102	Biolegend
CD73	BV605	344023	Biolegend
CD19	BV650	302238	Biolegend
CD39	BV785	328240	Biolegend
CD45RB	PE	310204	Biolegend
CD38	PE-Dazzle594	303538	Biolegend
CD3	PE-Cy5	300310	Biolegend
CD27	PE-Cy7	302838	Biolegend

Supplementary table 3: Flow cytometry panel for characterizing the T cells			
**Cell surface Marker**	**Fluorochrome**	**Catalog no.**	**Vendor**
CD8	FITC	344704	Biolegend
CCR7	PerCP-Cy5.5	353220	Biolegend
PD-1	APC	367406	Biolegend
CD3	AF700	300324	Biolegend
CD25	APC-Cy7	302614	Biolegend
CCR4	BV421	359414	Biolegend
Zombie	Amcyan	423102	Biolegend
CD127	BV605	351334	Biolegend
CCR6	BV650	353426	Biolegend
CXCR3	BV711	353732	Biolegend
CD45RA	BV785	304140	Biolegend
TCRgd	PE	MHGD04	Invitrogen
CD56	PE-Dazzle594	362544	Biolegend
CD4	PE-Cy5	317412	Biolegend
CXCR5	PE-Cy7	356924	Biolegend

Supplementary table 4: Flow cytometry panel for identifying the HA+ B cell subsets			
**Cell surface Marker**	**Fluorochrome**	**Catalog no.**	**Vendor**
CD19	BV650	302238	Biolegend
CD3	BV605	317322	Biolegend
CD27	PE-Cy7	302838	Biolegend
CD38	PE-Dazzle594	303538	Biolegend
IgD	APC/cy7	348218	Biolegend
Zombie	Amcyan	423102	Biolegend
Streptavidin	PE	12-4317-87	Invitrogen
Streptavidin	APC	17-4317-82	Invitrogen

Supplementary table 5: Coating antigens for ELISA using LAIV 2019-20 vaccine strained matched HA			
**Protein Type**	**Coating antigen**	**Catalog no.**	**Vendor**
LAIV 2019-2000 HA (vaccine strain matched)	A/Brisbane/02/2018 (H1N1)	IT-003-0011ΔTMp	Immune Technology
	A/Kansas/14/2017 (H3N2)	IT-003-00436p	Immune Technology
	B/Phuket/3073/2013	IT-003-B11ΔTMp	Immune Technology
	B/Colorado/06/2017	IT-003-B21△TMP	Immune Technology

Supplementary Table 6. Protein microarray influenza strains					
**Name**	**Strain**	**Strain year**	**Protein**	**Vendor**	**Catalog no.**
A/Cambodia/e0826360/2020	H3N2	2020	HA	Sino Biological	40789-V08H1
A/Cambodia/e0826360/2020	H3N2	2020	HA	Sino Biological	40789-V08H
A/Wisconsin/588/2019 / A/Victoria/2570/2019	H1N1	2019	HA	Sino Biological	40787-V08H1
A/Guangdong-Maonan/SWL1536/2019) / (A/Hawaii/70/2019)	H1N1	2019	HA	Sino Biological	40717-V08H
A/Hong Kong/2671/2019	H3N2	2019	HA	Sino Biological	40721-V08H
B/Washington/02/2019	B	2019	HA	Sino Biological	40722-V08H
A/Brisbane/02/2018	H1N1	2018	HA	Sino Biological	40719-V08H
A/Kansas/14/2017	H3N2	2017	HA	Sino Biological	40720-V08H
B/Colorado/06/2017	B	2017	HA	Sino Biological	40581-V08H
A/Singapore/INFIMH-16-0019/2016	H3N2	2016	HA	Sino Biological	40580-V08H
A/Michigan/45/2015	H1N1	2015	HA	Sino Biological	40567-V08H1
A/Michigan/45/2015	H1N1	2015	NA	Sino Biological	40568-V07H
A/Missouri/09/2014	H3N2	2014	HA	Sino Biological	40494-V08B

A/Switzerland/9715293/2013	H3N2	2013	HA	Sino Biological	40497-V08B
A/pigeon/Shanghai/S1069/2013	H7N9	2013	HA	Sino Biological	40106-V08H
A/Anhui/1/2013	H7N9	2013	HA	Sino Biological	40103-V08H
A/Shanghai/1/2013	H7N9	2013	HA	Sino Biological	40104-V08H
A/Shanghai/2/2013	H7N9	2013	HA	Sino Biological	40239-V08H
A/Hangzhou/1/2013	H7N9	2013	HA	Sino Biological	40105-V08H
A/JiangxiDonghu/346/2013	H10N8	2013	HA	Sino Biological	40359-V08B
B/Phuket/3073/2013	B	2013	HA	Sino Biological	40498-V08H1
A/Anhui/1/2013	H7N9	2013	NA	Sino Biological	40108-V07H
A/Shanghai/1/2013	H7N9	2013	NA	Sino Biological	40109-V07H
B/Phuket/3073/2013	B	2013	NA	Sino Biological	40502-V07B
A/Texas/50/2012	H3N2	2012	HA	Sino Biological	40354-V08B
B/Wisconsin/02/2012	B	2012	HA	Sino Biological	40462-V08H1
B/Utah/02/2012	B	2012	HA	Sino Biological	40463-V08H1
A/HongKong/CUHK31987/2011	H3N2	2011	HA	Sino Biological	40146-V08B
A/Victoria/361/2011	H3N2	2011	HA	Sino Biological	40145-V08B
A/chicken/Guangdong/C273/2011	H6N2	2011	HA	Sino Biological	40398-V08B
A/Hubei/1/2011	H5N1	2011	NA	Sino Biological	40018-V07H
A/barnswallow/HongKong/D101161/2010	H5N1	2010	HA	Sino Biological	40160-V08B
A/Hubei/1/2010	H5N1	2010	HA	Sino Biological	40015-V08H
A/flatfacedbat/Peru/033/2010	H18N11	2010	HA	Sino Biological	40324-V08B
B/Massachusetts/03/2010	B	2010	HA	Sino Biological	40191-V08H1
A/California/06/2009	H1N1	2009	HA	Sino Biological	40350-V08B
A/Texas/05/2009	H1N1	2009	HA	Sino Biological	40006-V08H
A/England/195/2009	H1N1	2009	HA	Sino Biological	40005-V08H
A/Beijing/22808/2009	H1N1	2009	HA	Sino Biological	40035-V08H
A/Ohio/07/2009	H1N1	2009	HA	Sino Biological	40007-V08H
A/NewYork/18/2009	H1N1	2009	HA	Sino Biological	40009-V08H
A/California/07/2009 HA1+HA2 His tag	H1N1	2009	HA	Sino Biological	11085-V08H
A/California/04/2009	H1N1	2009	HA	Sino Biological	11055-V08H
A/Hanoi/EL201/2009	H3N2	2009	HA	Sino Biological	40490-V08B
A/Victoria/208/2009	H3N2	2009	HA	Sino Biological	40151-V08B
A/Perth/16/2009	H3N2	2009	HA	Sino Biological	40043-V08H
A/GuangdongLuohu/1256/2009	H3N2	2009	HA	Sino Biological	40152-V08B
A/Perth/16/2009	H3N2	2009	HA	Sino Biological	40043-V08B1
A/duck/Hunan/819/2009	H4N2	2009	HA	Sino Biological	40390-V08B
A/Egypt/N05056/2009	H5N1	2009	HA	Sino Biological	11702-V08H
A/HongKong/35820/2009	H9N2	2009	HA	Sino Biological	40174-V08B
A/littleyellowshoulderedbat/Guatemala/164/2009	H17N10	2009	HA	Sino Biological	40323-V08B

A/California/04/2009	H1N1	2009	NA	Sino Biological	11058-V07B
A/California/04/2009	H1N1	2009	NA	Sino Biological	11058-V08B
A/California/07/2009	H1N1	2009	NP	Sino Biological	40205-V08B
H1N1A/California/04/09	H1N1	2009	NS1	eEnzyme	IA-NS1-021P
A/Hanoi/EL134/2008	H3N2	2008	HA	Sino Biological	40489-V08B
A/chicken/Vietnam/NCVD016/2008	H5N1	2008	HA	Sino Biological	40158-V08B
A/Vietnam/UT31413II/2008	H5N1	2008	HA	Sino Biological	40022-V08H
A/Cambodia/S1211394/2008	H5N1	2008	HA	Sino Biological	40026-V08H
A/HongKong/3239/2008	H9N2	2008	HA	Sino Biological	40178-V08B
B/Brisbane/60/2008	B	2008	HA	Sino Biological	40016-V08H1
B/Brisbane/60/2008	B	2008	NA	Sino Biological	40203-VNAHC
A/Brisbane/59/2007	H1N1	2007	HA	Sino Biological	11052-V08H
A/Ohio/UR060991/2007	H1N1	2007	HA	Sino Biological	11687-V08H
A/Brisbane/10/2007	H3N2	2007	HA	Sino Biological	11056-V08H
A/commonmagpie/HongKong/5052/2007	H5N1	2007	HA	Sino Biological	40044-V08H
A/Cambodia/R0405050/2007	H5N1	2007	HA	Sino Biological	11710-V08H
A/Egypt/2321NAMRU3/2007	H5N1	2007	HA	Sino Biological	11697-V08H
A/northernshoveler/California/HKWF115/2007	H6N1	2007	HA	Sino Biological	11723-V08H
A/mallard/Minnesota/SG00194/2007	H10N3	2007	HA	Sino Biological	40184-V08B
A/bluewingedteal/Louisiana/SG00073/2007	H10N7	2007	HA	Sino Biological	40433-V08B
A/thickbilledmurre/Newfoundland/031/2007	H11N2	2007	HA	Sino Biological	40187-V08B
B/Brisbane/3/2007	B	2007	HA	Sino Biological	40431-V08H1
A/Egypt/2321NAMRU3/2007	H5N1	2007	NA	Sino Biological	40045-VNAHC
A/SolomonIslands/3/2006	H1N1	2006	HA	Sino Biological	11708-V08H
A/Xinjiang/1/2006	H5N1	2006	HA	Sino Biological	40004-V08H
A/chicken/India/NIV33487/2006	H5N1	2006	HA	Sino Biological	11712-V08H
A/commonmagpie/HongKong/2256/2006	H5N1	2006	HA	Sino Biological	11700-V08H
A/goose/Guiyang/337/2006	H5N1	2006	HA	Sino Biological	11690-V08H
A/mallard/Netherlands/33/2006	H7N8	2006	HA	Sino Biological	40172-V08B
A/ruddyturnstone/NewJersey/563/2006	H7N2	2006	HA	Sino Biological	40170-V08B
B/Florida/4/2006	B	2006	HA	Sino Biological	11053-V08H1
B/Florida/4/2006	B	2006	NP	Sino Biological	40438-V08B
A/Canada/720/2005	H2N2	2005	HA	Sino Biological	11688-V08H
A/Wisconsin/67/2005	H3N2	2005	HA	Sino Biological	11972-V08H
A/barheadedgoose/Qinghai/1A/2005	H5N1	2005	HA	Sino Biological	40117-V08B
A/Anhui/1/2005	H5N1	2005	HA	Sino Biological	11048-V08H4
A/whooperswan/Mongolia/244/2005	H5N1	2005	HA	Sino Biological	11709-V08H
B/Ohio/01/2005	B	2005	HA	Sino Biological	40460-V08H1
A/Babol/36/2005	H3N2	2005	NA	Sino Biological	40017-V07H

A/Anhui/1/2005	H5N1	2005	NA	Sino Biological	11676-VNAHC1
A/California/7/2004	H3N2	2004	HA	Sino Biological	40118-V08B
A/chicken/Jilin/9/2004	H5N1	2004	HA	Sino Biological	40372-V08B
A/chicken/Yamaguchi/7/2004	H5N1	2004	HA	Sino Biological	40088-V08H
B/Florida/07/2004	B	2004	HA	Sino Biological	40432-V08H1
B/Malaysia/2506/2004	B	2004	HA	Sino Biological	11716-V08H1
A/Thailand/1(KAN-1)/2004	H5N1	2004	NA	Sino Biological	40064-V07H
A/Wyoming/03/2003	H3N2	2003	HA	Sino Biological	11715-V08H
A/HongKong/213/2003	H5N1	2003	HA	Sino Biological	11713-V08H
A/chicken/Netherlands/1/2003	H7N7	2003	HA	Sino Biological	11212-V08B
A/Netherlands/219/2003	H7N7	2003	HA	Sino Biological	11082-V08B
A/shorebird/Delaware/261/2003	H9N5	2003	HA	Sino Biological	40181-V08B
A/Philippines/472/2002	H3N2	2002	HA	Sino Biological	40487-V08B
A/Fujian/411/2002	H3N2	2002	HA	Sino Biological	40488-V08B
A/mallard/Ohio/657/2002	H4N6	2002	HA	Sino Biological	11714-V08H
A/duck/Hunan/795/2002	H5N1	2002	HA	Sino Biological	11698-V08H
A/turkey/Italy/214845/2002	H7N3	2002	HA	Sino Biological	40128-V08B
A/duck/Yangzhou/906/2002	H11N2	2002	HA	Sino Biological	11705-V08H
A/duck/Shantou/83/2000	H6N2	2000	HA	Sino Biological	40166-V08B
A/blackheadedgull/Netherlands/1/2000	H13N8	2000	HA	Sino Biological	11721-V08H
B/Victoria/504/2000	B	2000	HA	Sino Biological	40391-V08H
A/NewCaledonia/20/1999	H1N1	1999	HA	Sino Biological	11683-V08H
A/Moscow/10/1999	H3N2	1999	HA	Sino Biological	40154-V08B
A/swine/Ontario/019111/1999	H4N6	1999	HA	Sino Biological	11706-V08H
A/HongKong/1073/1999	H9N2	1999	HA	Sino Biological	11229-V08H
A/guineafowl/HongKong/WF10/1999	H9N2	1999	HA	Sino Biological	11719-V08H
A/blackheadedgull/Sweden/1/1999	H13N6	1999	HA	Sino Biological	40190-V08B
A/blackheadedgull/Sweden/5/1999	H16N3	1999	HA	Sino Biological	11711-V08H
A/swine/Belgium/1/1998	H1N1	1998	HA	Sino Biological	40393-V08B
A/mallard/Ohio/217/1998	H6N8	1998	HA	Sino Biological	40168-V08B
A/Sydney/5/1997	H3N2	1997	HA	Sino Biological	40149-V08B
A/duck/HongKong/P46/1997	H5N1	1997	HA	Sino Biological	40001-V08H
A/HongKong/483/1997	H5N1	1997	HA	Sino Biological	11689-V08H
A/chicken/HongKong/G9/1997	H9N2	1997	HA	Sino Biological	40036-V08H
A/goose/Guangdong/1/1996	H5N1	1996	HA	Sino Biological	40024-V08B
A/Beijing/262/1995	H1N1	1995	HA	Sino Biological	40133-V08B
A/Netherlands/178/1995	H3N2	1995	HA	Sino Biological	40486-V08B
A/Nanchang/933/1995	H3N2	1995	HA	Sino Biological	40485-V08B
A/Johannesburg/33/1994	H3N2	1994	HA	Sino Biological	40476-V08B

A/Texas/36/1991	H1N1	1991	HA	Sino Biological	40132-V08B
A/greenwingedteal/ALB/199/1991	H12N5	1991	HA	Sino Biological	11718-V08H
B/Yamagata/16/1988	B	1988	HA	Sino Biological	40157-V08H1
B/Victoria/02/1987	B	1987	HA	Sino Biological	40163-V08H
A/duck/NewZealand/76/1984	H9N1	1984	HA	Sino Biological	40179-V08B
A/mallardduck/Alberta/342/1983	H12N1	1983	HA	Sino Biological	40029-V08H
A/australianshelduck/WesternAustralia/1756/1983	H15N2	1983	HA	Sino Biological	40193-V08B
A/duck/Australia/341/1983	H15N8	1983	HA	Sino Biological	11720-V08H
A/mallard/Astrakhan/263/1982	H14N5	1982	HA	Sino Biological	40192-V08B
A/pintailduck/Alberta/115/1979	H8N4	1979	HA	Sino Biological	11722-V08H
A/duck/HongKong/562/1979	H10N9	1979	HA	Sino Biological	40028-V08H
A/duck/HongKong/786/1979	H10N3	1979	HA	Sino Biological	11693-V08H
A/duck/HongKong/448/1978	H9N2	1978	HA	Sino Biological	40003-V08B
A/USSR/90/1977	H1N1	1977	HA	Sino Biological	40134-V08B
A/mallardduck/Alberta/299/1977	H4N4	1977	HA	Sino Biological	40008-V08H
A/chicken/HongKong/17/1977	H6N4	1977	HA	Sino Biological	40027-V08H
A/mallard/Alberta/294/1977	H11N9	1977	HA	Sino Biological	11704-V08H
A/USSR/90/1977	H1N1	1977	NA	Sino Biological	40197-V07H
A/NewJersey/8/1976	H1N1	1976	HA	Sino Biological	40392-V08B
A/chicken/Alabama/1/1975	H4N8	1975	HA	Sino Biological	40025-V08H
B/HongKong/05/1973	B	1973	HA	Sino Biological	40461-V08H1
A/Aichi/2/1968	H3N2	1968	HA	Sino Biological	11707-V08H
A/Aichi/2/1968	H3N2	1968	NA	Sino Biological	40199-V07H
A/Aichi/2/1968	H3N2	1968	M1	Sino Biological	40215-V07E
A/Hong Kong/1/1968	H3N2	1968	NP	Sino Biological	40208-V08B
A/Aichi/2/1968	H3N2	1968	NP	Sino Biological	40207-V08B
A/Guiyang/1/1957	H2N2	1957	HA	Sino Biological	40119-V08B
A/Japan/305/1957	H2N2	1957	HA	Sino Biological	11088-V08H
A/Albany/12/1951	H1N1	1951	HA	Sino Biological	40464-V08B
A/PuertoRico/8/1934	H1N1	1934	HA	Sino Biological	11684-V08H
A/PuertoRico/8/1934	H1N1	1934	NA	Sino Biological	40196-VNAHC
A/Puerto Rico/8/34/Mount Sinai	H1N1	1934	M1	Sino Biological	40010-V07E
A/Puerto Rico/8/34/Mount Sinai	H1N1	1934	NP	Sino Biological	11675-V08B
A/Puerto Rico/8/34/Mount Sinai	H1N1	1934	NS1	Sino Biological	40011-V07E
A/Puerto Rico/8/34/Mount Sinai	H1N1	1934	NS2	Sino Biological	40012-VNAE
A/WSN/1933	H1N1	1933	HA	Sino Biological	11692-V08H
A/NewYork/1/1918	H1N1	1918	HA	Sino Biological	40090-V08B
A/BrevigMission/1/1918	H1N1	1918	HA	Sino Biological	11068-V08H
A/Brevig Mission/1/1918	H1N1	1918	M1	Sino Biological	40211-V07E
